# CeRNA regulatory network-based analysis to study the roles of noncoding RNAs in the pathogenesis of intrahepatic cholangiocellular carcinoma

**DOI:** 10.18632/aging.102634

**Published:** 2020-01-19

**Authors:** Weiyu Xu, Si Yu, Jianping Xiong, Junyu Long, Yongchang Zheng, Xinting Sang

**Affiliations:** 1 Department of General Surgery, Beijing Friendship Hospital, Capital Medical University, Xi-Cheng, Beijing 100050, People's Republic of China; 2 Department of Liver Surgery, Peking Union Medical College Hospital, Chinese Academy of Medical Sciences and Peking Union Medical College, Wangfujing, Beijing 100730, People's Republic of China; 3 Department of Interventional Radiology, Beijing Friendship Hospital, Capital Medical University, Xi-Cheng, Beijing 100050, People's Republic of China

**Keywords:** intrahepatic cholangiocarcinoma, ceRNA, ceRNA regulatory network, biomarkers, prognosis

## Abstract

To explore and understand the competitive mechanism of ceRNAs in intrahepatic cholangiocarcinoma (ICC), we used bioinformatics analysis methods to construct an ICC-related ceRNA regulatory network (ceRNET), which contained 340 lncRNA-miRNA-mRNA regulatory relationships based on the RNA expression datasets in the NCBI GEO database. We identified the core regulatory pathway RP11-328K4.1-hsa-miR-27a-3p-PROS1, which is related to ICC, for further validation by molecular biology assays. GO analysis of 44 differentially expressed mRNAs in ceRNET revealed that they were mainly enriched in biological processes including “negative regulation of epithelial cell proliferation” and "positive regulation of activated T lymphocyte proliferation.” KEGG analysis showed that they were mainly enriched in the “complement and coagulation cascade” pathway. The molecular biology assay showed that lncRNA RP11-328K4.1 expression was significantly lower in the cancerous tissues and peripheral plasma of ICC patients than in normal controls (p<0.05). In addition, hsa-miR-27a-3p was found to be significantly upregulated in the cancer tissues and peripheral plasma of ICC patients (p<0.05). Compared to normal controls, the expression of PROS1 mRNA was significantly downregulated in ICC patient cancer tissues (p<0.05) but not in peripheral plasma (p>0.05). Furthermore, ROC analysis revealed that RP11-328K4.1, hsa-miR-27a-3p, and PROS1 had significant diagnostic value in ICC. We concluded that the upregulation of lncRNA RP11-328K4.1, which might act as a miRNA sponge, exerts an antitumor effect in ICC by eliminating the inhibition of PROS1 mRNA expression by oncogenic miRNA hsa-miR-27a.

## INTRODUCTION

Intrahepatic cholangiocarcinoma (ICC) is an adenocarcinoma that originates from the intrahepatic secondary bile duct and its branch epithelium. ICC is anatomically different from the other two types of cholangiocarcinoma (CCA): perihilar cholangiocarcinoma (pCCA) and distal cholangiocarcinoma (dCCA) [1, 2]. The incidence of ICC accounts for 10%-15% of primary hepatic malignant tumors, only second to the incidence of primary hepatocellular carcinoma [1]. Statistics from recent years indicate that the morbidity and mortality rates of ICC continue to show an increasing trend globally [1, 3]. Due to a lack of obvious clinical symptoms and limited effective screening methods in the early stage of the disease, most ICC patients do not have the option of surgery at diagnosis; only 30%-40% of patients have the opportunity to get surgery after diagnosis [4, 5]. Patients who have not undergone surgery have an extremely poor prognosis, and few patients survive for more than three years. The 3-year survival rate of patients undergoing surgery is only 40%-50% [6]. Therefore, it is particularly urgent and necessary to better understand the molecular mechanisms underlying the pathogenesis and progression of ICC and to find potential biomarkers for diagnosis and prognosis as well as therapeutic targets for ICC.

Early research on the molecular mechanism of carcinogenesis has mainly focused on different protein-coding genes. With the development and popularization of high-throughput whole-genome sequencing technology, a variety of noncoding ribonucleic acids (ncRNA) of different lengths have been clearly found to play key regulatory roles in human carcinogenesis, including noncoding RNAs (ncRNAs), such as long noncoding RNA (lncRNA) and microRNA (miRNA) [7]. lncRNAs are a subset of noncoding transcripts of over 200 nucleotides in length, with little or no protein-coding ability, and they play key roles in a series of biological processes by regulating gene expression through mechanisms including transcription, splicing, and translation [8, 9]. Due to their greater tissue specificity, lncRNAs are more effective as biomarkers for the early diagnosis and screening of cancer patients [10]. Recent studies [11, 12] have found that lncRNAs may be potential diagnostic and prognostic biomarkers for CCA and that they may be related to the pathogenesis and progression of CCA. miRNAs are a class of endogenous small RNAs approximately 20-24 nucleotides in length that play various important regulatory functions within cells [13]. Each miRNA can regulate multiple target genes, and several miRNAs can regulate a single gene. Therefore, this complex regulatory network can regulate the expression of multiple genes through a single miRNA or specifically regulate the expression of a single gene through the combination of multiple miRNAs [14]. Similarly, some studies [15–19] also suggest that aberrantly expressed miRNAs can be used as diagnostic and prognostic markers for CCA that are closely associated with the pathogenesis, progression and metastasis of CCA. However, the underlying mechanisms of lncRNAs and miRNAs in CCA, especially in ICC, are not fully understood.

Recent studies have shown that lncRNAs, as competitive endogenous RNA (ceRNA) with miRNA response elements (MRE), can compete with mRNAs for binding with miRNAs, thus affecting gene expression [20–22]. Abnormal regulation of ceRNA is involved in multiple types of cancers, such as breast cancer, lung cancer, gastric cancer, colorectal cancer, hepatic cancer and CCA [23–29]. Mathematical modeling, informatics-based analysis and experimental validation have been used to describe the structure of ceRNA regulatory networks (ceRNETs) and their role in regulating cellular physiology under normal conditions and pathological conditions such as cancer [22]. Previous studies have thoroughly discussed the diagnostic and prognostic value of lncRNA-related ceRNETs and their pivotal role in the pathogenesis and progression of HCC. Gao M et al. [30] constructed ceRNETs of HCC-related lncRNAs (HOTAIR and MALAT1) by using bioinformatics methods. These networks predicted that MALAT1 and HOTAIR can act as miRNA sponges to inhibit hsa-miR-1 and hsa-miR-20a-5p, thereby removing the inhibition of the expression of cyclin D1, E2F1, EGFR, MYC, MET, NOS2A and VEGFA. Gene Ontology (GO) and Kyoto Encyclopedia of Genes and Genomes (KEGG) enrichment analyses of these seven HCC-related miRNA target genes indicates that MALAT1 and HOTAIR could promote cell growth, cell cycle progression and mitosis by involving in cell cycle, focal adhesion and disease progression pathways. Yan Y et al. [31] identified nine key lncRNAs in the overall ceRNET by constructing lncRNA-related ceRNETs (HCG18, AC021078.1, ENT-PD1-AS1, MCM3AP-AS1, GMDS-AS1, AC019080.1, AC245452.1, LINC00630 and AP000766.1). Functional enrichment analysis of coexpressed adjacent mRNAs revealed a close association with liver function and the pathogenesis of HCC. Further construction of a ceRNA subnet associated with HCC prognosis was done by screening 16 lncRNAs associated with HCC prognosis, which were further used for constructing a risk scoring model. According to the median risk score, the overall survival (OS) was significantly higher in the low-risk group than in the high-risk group (P = 8.31e-05). At present, studies on the pathogenesis of CCA by constructing ceRNETs and examining their role in the diagnosis, prognosis and treatment of CCA are relatively rare and lack depth. The limited CCA-related ceRNETs constructed by Song W et al. [29] contain 116 lncRNAs, 14 miRNAs and 59 mRNAs. Functional enrichment analysis revealed that these ncRNAs promote CCA progression mainly through the estrogen signaling pathway and MAPK. Meanwhile, seven lncRNAs with negative correlations with CCA prognosis and four lncRNAs with positive correlations with CCA prognosis were identified. However, CCA includes three types, ICC, pCCA and dCCA, which are significantly different in anatomical location, clinical manifestation, morphology and epidemiology. At present, the role of ceRNETs in ICC remains unclarified.

In this study, to determine the diagnostic, therapeutic, and prognostic value of lncRNA-related ceRNETs and their key role in the pathogenesis and progression of ICC, we performed integrated analysis of expression profile data on ICC-related lncRNA, miRNA and mRNA from National Center for Biotechnology Information Gene Expression Omnibus (NCBI GEO). Afterwards, we integrated these identified differential expression (DE) RNAs and constructed a lncRNA-miRNA-mRNA regulatory network. Moreover, functional enrichment analysis was performed on mRNA involved in ceRNET construction. Then, based on the ranking of each ceRNA in ceRNETs (including connectivity and log-fold change, logFC), their relationships (whether their direction of expression changes is consistent or opposite), and their mention in the results of previous studies in other tumors, we identified the most related core regulatory pathways of ICC. Finally, we experimentally validated the mRNA and corresponding protein in the core ceRNET regulatory pathway with ICC fresh tissues, blood, and paraffin sections. We also clinically validated the RNAs in the core ceRNET regulatory pathway with datasets from the NCBI GEO and TCGA. In combination with experimental results, clinical outcomes, and previous research, the mechanism of these noncoding RNAs and their constituent pathways in ICC was further discussed.

## RESULTS

### ICC-related differentially expressed lncRNAs, miRNAs and mRNAs based on GEO microarray chips

**Figure 1:**
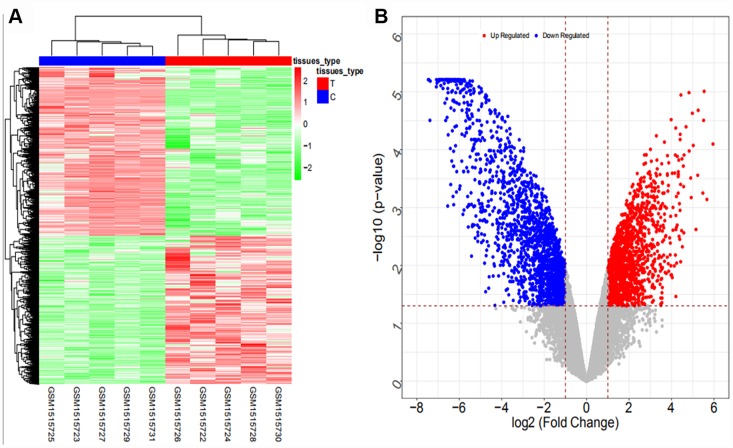
Heat map (**A**) and volcano map (**B**) of differentially expressed mRNAs in the GSE61850 dataset.

**Figure 2:**
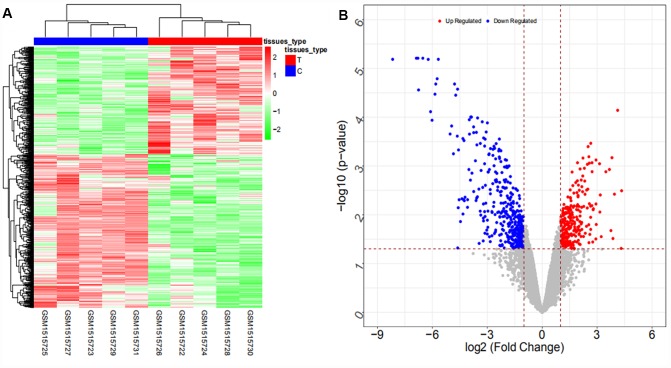
Heat map (**A**) and volcano map (**B**) of differentially expressed lncRNAs in the GSE61850 dataset.

**Figure 3:**
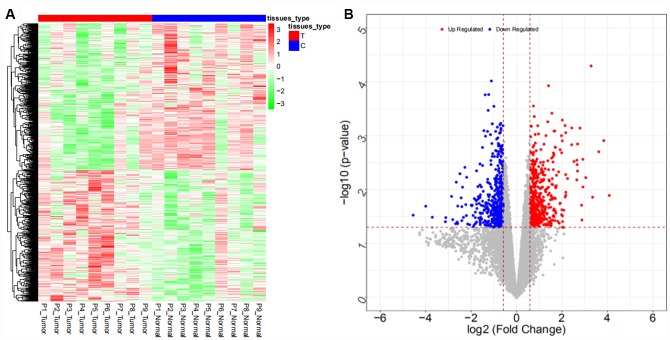
Heat map (**A**) and volcano map (**B**) of differentially expressed mRNAs in the GSE103909 dataset.

**Figure 4:**
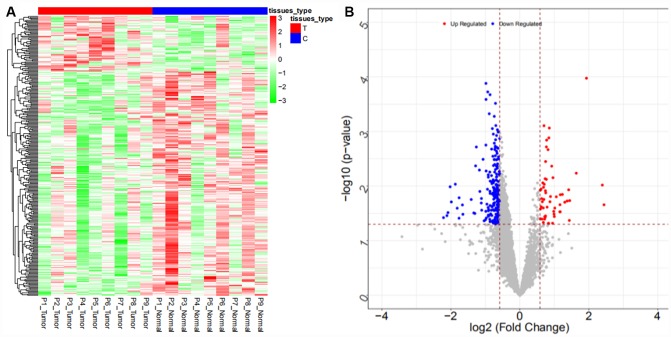
Heat map (**A**) and volcano map (**B**) of differentially expressed lncRNAs in the GSE103909 dataset.

**Figure 5:**
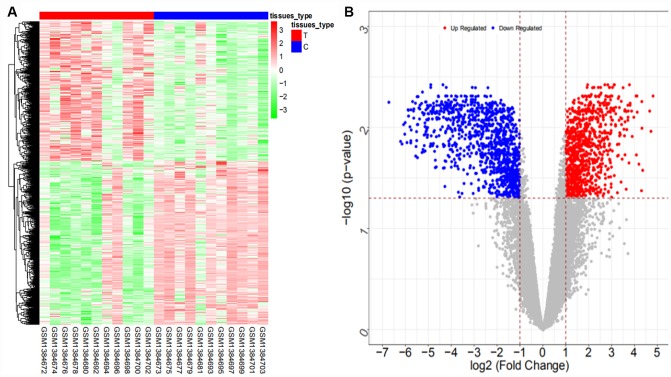
Heat map (**A**) and volcano map (**B**) of differentially expressed mRNAs in the GSE57555 dataset.

**Figure 6:**
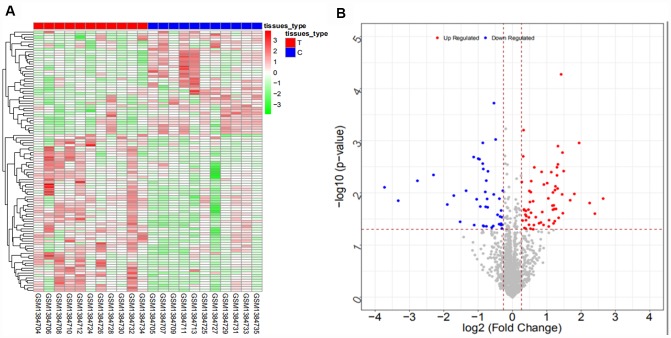
Heat map (**A**) and volcano map (**B**) of differentially expressed miRNAs in the GSE57555 dataset.

**Figure 7:**
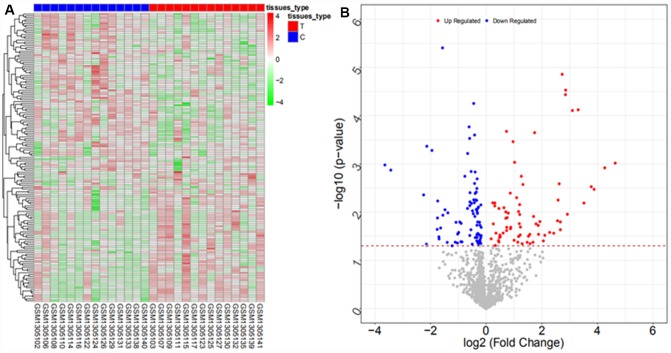
Heat map (**A**) and volcano map (**B**) of differentially expressed miRNAs in the GSE53992 dataset

**Figure 8:**
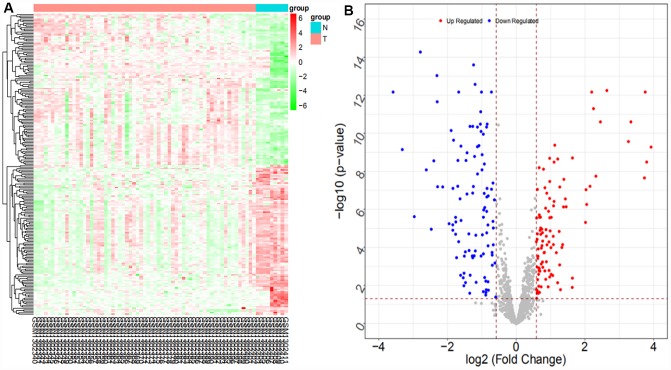
Heat map (**A**) and volcano map (**B**) of differentially expressed miRNAs in the GSE53870 dataset

**Figure 9:**
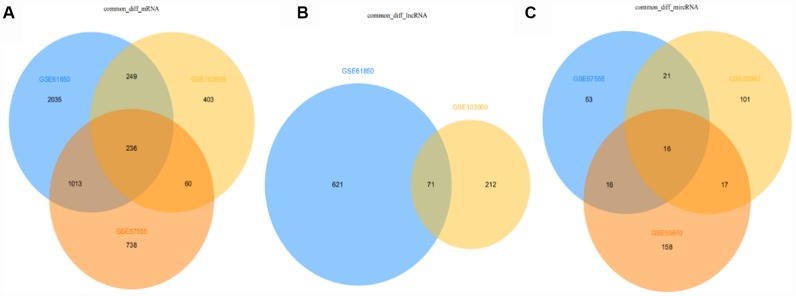
Venn diagram of differentially expressed mRNAs, lncRNAs, miRNAs in all datasets (from **A** to **B** to **C**: mRNA, lncRNA, miRNA).

From the GSE61850 dataset, a total of 3533 differentially expressed mRNAs (1650 upregulated and 1883 downregulated mRNAs) and 692 differentially expressed lncRNAs (286 upregulated and 406 downregulated lncRNAs) were obtained. The heat map and volcano map are shown in Figures 1 and 2, respectively (partial data are shown in Supplementary Tables 1 and 2):

From the GSE103909 dataset, a total of 948 differentially expressed mRNAs (447 upregulated and 501 downregulated mRNAs) and 283 differentially expressed lncRNAs (56 upregulated and 227 downregulated lncRNAs) were obtained. The heat map and volcano map are shown in Figures 3 and 4, respectively (partial data are shown in Supplementary Tables 3 and 4):

From the GSE57555 dataset, a total of 2047 differentially expressed mRNAs were obtained (942 upregulated and 1105 downregulated mRNAs), and 106 differentially expressed miRNAs (64 upregulated and 42 downregulated miRNAs) were obtained. The heat map and volcano map are shown in Figures 5, 6, respectively (partial data are shown in Supplementary Tables 5 and 6):

From the GSE53992 dataset, a total of 155 differentially expressed miRNAs (71 upregulated and 84 downregulated miRNAs) were obtained. The heat map and volcano map are shown in Figure 7 (partial data are shown in Supplementary Table 7):

From the GSE53870 dataset, a total of 207 differentially expressed miRNAs were obtained (104 upregulated and 103 downregulated). The heat map and volcano map are shown in Figure 8 (partial data are shown in Supplementary Table 8):

The intersections of differentially expressed mRNAs, differentially expressed lncRNAs, and differentially expressed miRNAs are shown in the Venn map (Figure 9), with a total of 236 consensus differentially expressed mRNAs, 71 consensus differentially expressed lncRNAs, and 16 consensus differentially expressed miRNAs. Some of the results are shown in Supplementary Tables 9–11.

### Coexpression analysis and miRNA target gene prediction analysis for constructing lncRNA-miRNA and miRNA-mRNA relationship pairs

Coexpression analysis and the intersection of GSE61850 and GSE103909 datasets revealed 1403 lncRNA-mRNA relationship pairs with synergistic expression, including 194 mRNAs and 54 lncRNAs (some results are shown in Supplementary Table 12). Coexpression analysis of the GSE57555 dataset revealed 1166 miRNA-mRNA inversely correlated relationship pairs, including 16 miRNAs and 220 mRNAs (some results are shown in Supplementary Table 13).

The online tool mirwalk2.0 was used to perform target gene prediction on the above 16 miRNAs. A total of 29,426 miRNA-mRNA relationship pairs were obtained (some results are shown in Supplementary Table 14). By intersecting with the miRNA-mRNA obtained in the first step, a total of 113 miRNA-mRNA relationship pairs were obtained, including 12 miRNAs and 58 mRNAs (detailed results are shown in Supplementary Table 15). A total of 54 lncRNAs and 16 miRNAs from the coexpression analysis were extracted for the prediction of miRNA-lncRNA binding sites by using the local software Miranda (v3.3a), which revealed 362 miRNA-lncRNA relationship pairs, including 16 miRNAs and 53 lncRNAs (some results are shown in Supplementary Table 16).

### Construction of a ceRNA regulation network (ceRNET) based on GEO chip databases

To further explore how lncRNAs regulates mRNA expression by binding to miRNAs in ICC, based on the miRNA-lncRNA and miRNA-mRNA relationship pairs obtained from the previous step and according to the premise that mutual ceRNAs with the same miRNA binding sites exist in the ceRNA network, we first screened the mRNAs and lncRNAs regulated by the same miRNA. Then, according to the consistent expression trend among ceRNAs and the synergistic expression relationships between mRNAs and lncRNAs, we finally obtained 340 pairs of lncRNA-miRNA-mRNA regulatory relationships, containing 44 mRNAs, 12 miRNAs and 24 lncRNAs (some of the results are shown in Supplementary Table 17).

**Figure 10:**
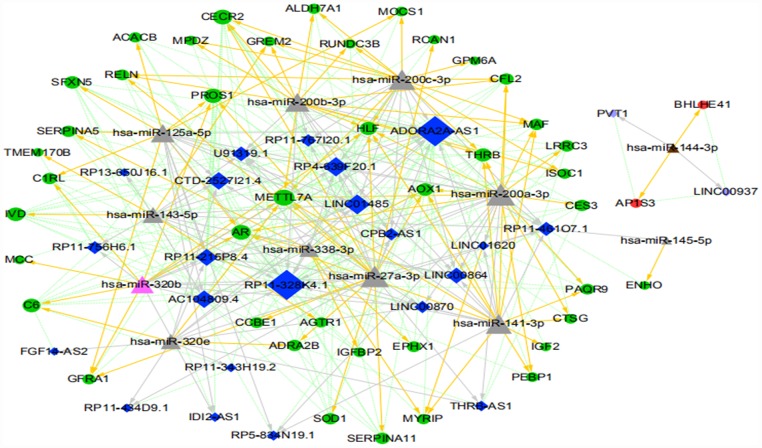
**The ICC-related ceRNA network map (green circles indicate the downregulated mRNAs, red circles indicate the upregulated mRNA, dark blue diamonds indicate the downregulated lncRNA, light purple diamonds indicate the upregulated lncRNA, pink triangles indicate the upregulated miRNA, brown triangles indicate the downregulated miRNA, and gray triangles indicate that upregulation or downregulation of miRNAs cannot be determined.** The gray arrow indicates the regulatory relationship between miRNA and lncRNA, the yellow arrow indicates the regulatory relationship between miRNA and mRNA, and the green dotted line indicates the synergistic expression relationship between lncRNA and mRNA).

Cytoscape 3.4.0 was used to construct a ceRNA network for the 340 lncRNA-miRNA-mRNA regulatory relationships obtained above. We also determined the upregulation and downregulation of these nodes (some results are shown in Supplementary Table 18). The ceRNA network is shown in Figure 10.

### GO and KEGG pathway enrichment analyses of differentially expressed mRNAs in the constructed ICC-related ceRNETs

**Figure 11:**
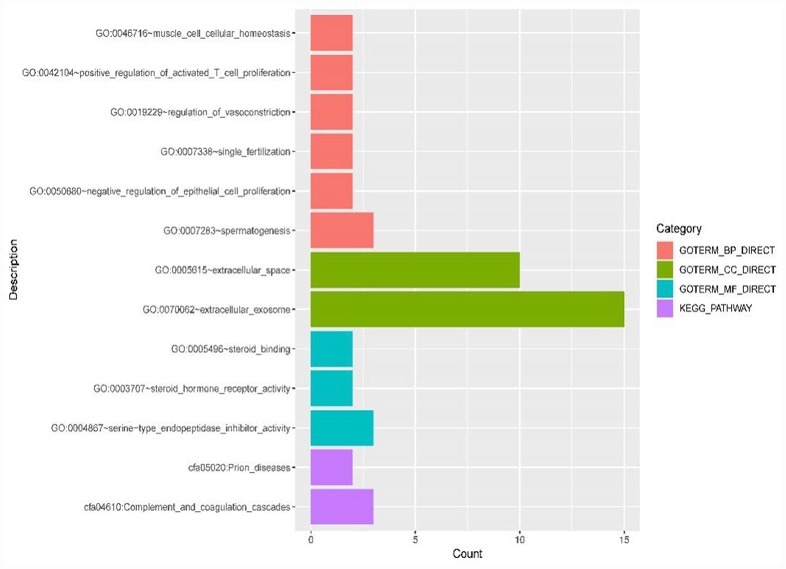
**GO terms (including biological process (BP), cellular component (CC) and molecular function (MF)) and KEGG pathways involved in the construction of ICC-related ceRNETs of 44 DE mRNAs.**

Functional enrichment analysis was performed on differentially expressed mRNA in the constructed ICC-related ceRNETs (shown in Figure 11 and Table 1). GO analysis revealed that the biological processes associated with differentially expressed mRNAs and tumors were mainly involved in the negative regulation of epithelial cell proliferation and the positive regulation of activated T cell proliferation. KEGG analysis revealed that differentially expressed mRNAs were mainly involved in the complement and coagulation cascade pathways.

**Table 1:** Specific DE mRNAs enriched in each GO term and KEGG pathway in ICC-related ceRNA networks.

**Category**	**Term**	**Count**	**Genes**	**PValue**
GOTERM_BP	muscle cell cellular homeostasis	2	CFL2, SOD1	0.033571
GOTERM_BP	positive regulation of activated T cell proliferation	2	IGF2, IGFBP2	0.035927
GOTERM_BP	regulation of vasoconstriction	2	AGTR1, ADRA2B	0.03121
GOTERM_BP	single fertilization	2	AR, CECR2	0.052263
GOTERM_BP	negative regulation of epithelial cell proliferation	2	AR, MCC	0.09083
GOTERM_BP	spermatogenesis	3	AR, SERPINA5, SOD1	0.058154
GOTERM_CC	extracellular space	10	SERPINA11, CFL2, SERPINA5, CCBE1, C1RL, RELN, IGF2, IGFBP2, SOD1, GREM2	1.36E-04
GOTERM_CC	extracellular exosome	15	THRB, C6, IGF2, ISOC1, METTL7A, SOD1, ALDH7A1, GPM6A, SERPINA5, CFL2, C1RL, GFRA1, PEBP1, IGFBP2, PROS1	2.97E-04
GOTERM_MF	steroid binding	2	AR, PAQR9	0.024354
GOTERM_MF	steroid hormone receptor activity	2	THRB, PAQR9	0.094041
GOTERM_MF	serine-type endopeptidase inhibitor activity	3	SERPINA11, SERPINA5, PEBP1	0.01002
KEGG_PATHWAY	Prion diseases	2	C6, SOD1	0.090786
KEGG_PATHWAY	Complement and coagulation cascades	3	SERPINA5, C6, PROS1	0.017377

Meanwhile, the Cytoscape plugin CytoNCA was used to analyze the node connectivity of the network, with unweighted parameters. The node size in the figure indicates the degree of connectivity in the network: the larger the node, the higher the degree of connectivity (some of the data are shown in Supplementary Table 19 for details). Based on the ranking of network nodes, the reproducibility of expression trends in several GEO datasets, the logFC and ceRNA relationship, and previous outcomes in other types of tumors, the following ceRNA regulatory relationships were finally chosen for further validation: RP11-328K4.1-hsa-miR-27a-3p-PROS1; RP11-328K4.1-hsa-miR-27a-3p-METTL7A; RP11-328K4.1-hsa-miR-200a-3p-METTL7A; RP11-328K4.1-hsa-miR-200a-3p-CECR2; ADORA2A-AS1-hsa-miR-200b-3p-CECR2; ADORA2A-AS1-hsa-miR-27a-3p-PROS1; ADORA2A-AS1-hsa-miR-200c-3p-CECR2; LINC01485-hsa-miR-200c-3p-CECR2; LINC01485-hsa-miR-200b-3p-METTL7A; and LINC01485-hsa-miR-200b-3p-CECR2.

**Figure 12:**
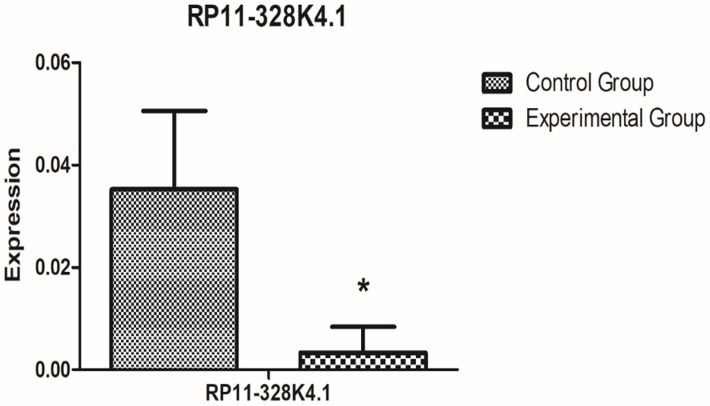
**qRT-PCR analysis showed the difference in lncRNA RP11-328K4.1 expression between the experimental group and control group in ICC fresh tissue samples after normalization to internal controls.** RP11-328K4.1 was normalized to β-actin.

After comprehensive analysis, we finally obtained the ICC-related core regulatory pathway, RP11-328K4.1-hsa-miR-27a-3p-PROS1, which was further validated in relevant fresh tissue, blood samples and paraffin sections. The results are shown as follows: The expression of lncRNA RP11-328K4.1 in 10 pairs of fresh ICC cancer and adjacent paracancerous tissues is shown in Figure 12. The expression of lncRNA RP11-328K4.1 was significantly decreased in the ICC experimental group (cancer tissue) compared to that in the control group (paracancerous tissue) (P = 0.000007).

**Figure 13:**
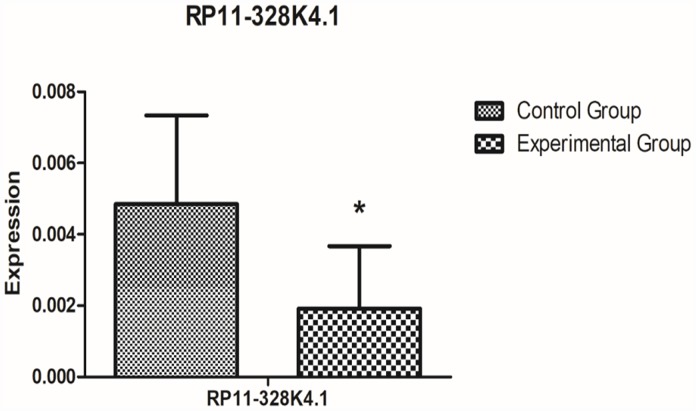
**qRT-PCR analysis showed the difference in lncRNA RP11-328K4.1 expression between the experimental group and control group in peripheral plasma samples after normalization to internal controls.** RP11-328K4.1 was normalized to β-actin.

The expression level of lncRNA RP11-328K4.1 in the peripheral plasma of 10 ICC patients and 10 healthy subjects is shown in Figure 13. The expression of lncRNA RP11-328K4.1 was significantly decreased in the experimental group (peripheral plasma of ICC patients) compared to that in the control group (peripheral plasma of healthy controls) (P=0.036093).

The expression levels of hsa-miR-27a-3p in 10 pairs of fresh ICC cancer and adjacent tissues are shown in Supplementary Figure 1. The expression level of hsa-miR-27a-3p was significantly higher in the experimental group (ICC cancer tissue) than that in the control group (paracancerous tissues) (P = 0.00016).

The expression levels of hsa-miR-27a-3p in the peripheral plasma of 10 ICC patients and 10 healthy subjects are shown in Supplementary Figure 2 below. The expression of hsa-miR-27a-3p was significantly higher in the experimental group (peripheral plasma of ICC patients) than that in the control group (peripheral plasma of healthy subjects) (P=0.04942034).

The expression levels of PROS1 mRNA in 10 pairs of fresh ICC cancer and paracancerous tissues are shown in Supplementary Figure 3. The expression of PROS1 mRNA was significantly decreased in the experimental group (ICC cancer tissues) compared with that in the control group (paracancerous tissues) (P = 0.006611).

The expression levels of PROS1 mRNA in the peripheral plasma of 10 ICC patients and 10 healthy subjects are shown in Supplementary Figure 4. The expression of PROS1 mRNA was decreased in the experimental group (peripheral plasma of ICC patients) compared to that in the control group (peripheral plasma of healthy subjects), however, the difference was not statistically significant (P=0.171259).

### Western Blot (WB) assay to detect protein corresponding to PROS1 mRNA in tissues

The expression levels of the protein corresponding to PROS1 mRNA in 10 pairs of fresh ICC cancer and adjacent noncancer tissues are shown in Supplementary Figures 5, 6. The expression of protein corresponding to PROS1 mRNA was lower in the experimental group (ICC cancer tissue) than that in the control group (paracancerous tissues), however, the difference was not statistically significant (P = 0.668353048).

### WB assay to detect protein corresponding to PROS1 mRNA in plasma

The expression levels of the protein corresponding to PROS1 mRNA in the peripheral plasma of 10 ICC patients and the peripheral plasma of 10 healthy subjects are shown in Supplementary Figures 7 and 8. The expression of protein corresponding to PROS1 mRNA was increased in the experimental group (peripheral plasma of ICC patients) compared to that in the control group (peripheral plasma of healthy subjects), however, the difference was not statistically significant (P=0.597799476).

### Immunohistochemistry (IHC) results of PROS1 expression in paraffin sections from ICC patients

In this study, we performed IHC on paraffin sections from 88 ICC patients. The median age of patients was 62 years (range: 30-83 years). There were 52 males and 36 females, with a male:female ratio of 1.75:1. A total of 56 patients underwent radical surgery, accounting for 63.6% of the total number of patients. The detailed data are shown in Table 2. The IHC results of PROS1 staining in cancer tissues and adjacent normal tissues were analyzed and are shown in Supplementary Figure 9A–9D: PROS1 showed positive staining in the cytoplasm of ICC cancer tissues, and the staining intensity could be divided into high, medium and low degrees. However, the expression of PROS1 in the cytoplasm of normal adjacent tissue was nearly negative.

**Table 2:** The baseline characteristics and IHC of 88 ICC patients receiving surgery.

**Characteristics**	**Number of patients (*n*=154)**	**Characteristics**	**Number of patients (*n*=154)**
Age (year)	62 (30-83)	yes	1(1.1)
≤ 60	34 (38.6)	jaundice	
> 60	54 (61.4)	no	71 (80.7)
Gender		yes	17 (19.3)
male	52 (59.1)	Blood type	
female	36 (40.9)	A	27 (30.7)
smoking		B	33 (37.5)
no	59(67.1)	AB	6 (6.8)
yes	29(32.9)	O	22 (25.0)
alcohol		GGT	270.1(12-2769)
no	71(80.7)	≤50	24 (27.3)
yes	17(19.3)	>50	64 (62.7)
BMI	24.10 (16.9-32.6)	differentiation	
<18.5	1(1.1)	Poorly differentiated	29 (33.0)
≥18.5 and <24	42(47.8)	Moderately-well	59 (67.0)
24	45(51.1)	differentiated	
gallstone		Margin status	
no	74 (84.1)	negative	56 (63.6)
yes	14 (15.9)	positive	32 (36.4)
cholangiolithiasis		Largest tumor diameter (cm)	4.83(1.0-14)
no	80(91)	≤ 5	53 (60.2)
yes	8(9)	> 5	35 (39.8)
cholecystitis		T stage	
no	70(79.5)	Tis-T1a	21 (23.9)
yes	18(20.5)	T1b	18 (20.5)
diabetes		T2	16(18.2)
no	69 (78.4)	T3	15 (17.1)
yes	19 (21.6)	T4	18 (20.5)
hypertension		N stage	
no	61(69.3)	0 stage	62 (70.5)
yes	27(30.7)	1 stage	26 (29.5)
Fatty liver		M stage	
no	86(97.7)	no	73 (83.0)
yes	2(2.3)	yes	15 (17.0)
cirrhosis		TNM stage	
no	82(93.2)	1A-IB	31 (35.3)
yes	6(6.8)	II	10 (11.4)
HBV		IIIA-IIIB	33 (37.5)
no	77(87.5)	IV	14 (15.9)
yes	11(12.5)	CA199 (U/ml)	1829.2 (0.5-28411)
HCV		≤ 39	29 (40.0)
no	87(98.9)	> 39	59 (60.0)
total bilirubin (umol/L)	39.9 (5.9-420)	28-35	9 (10.2)
≤17.1	58 (65.9)	> 35	79 (89.8)
> 17.1	30 (34.1)	AFP(ug/L)	20.3 (0.6-1091)
albumin level (g/L)	41.5 (29.0-51.0)	≤25	83 (94.3)
<28	0 (0.0)	>25	5 (5.7)

### Receiver Operating Characteristic (ROC) analysis of RP11-328K4.1, hsa-miR-27a-3p and PROS1

**Figure 14:**
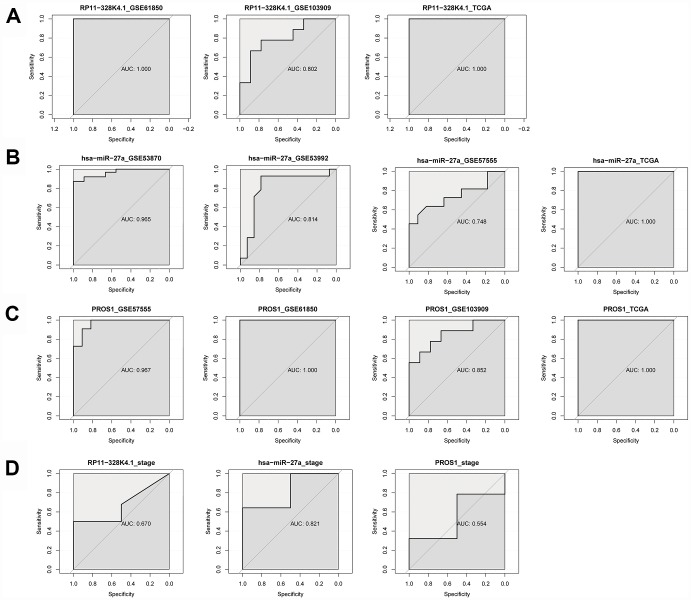
(**A**–**C**) ROC analysis of RP11-328K4.1, hsa-miR-27a-3p, and PROS1 in ICC tumor tissues and matched adjacent nontumor tissues. (**D**) ROC analysis of RP11-328K4.1, hsa-miR-27a-3p, and PROS1 in early ICC and advanced ICC.

ROC curves indicated that RP11-328K4.1, hsa-miR-27a-3p and PROS1 exhibited great diagnostic efficiency in ICC tumor tissues and nontumor tissues (Figure 14A–14C). The areas under the ROC curve (AUCs) of RP11-328K4.1 were 1.000, 0.802, and 1.000 in GSE61850, GSE103909, and TCGA, respectively. The AUCs of hsa-miR-27a-3p were 0.965, 0.814, 0.748 and 1.000 in GSE53870, GSE53992, GSE57555, and TCGA, respectively. The AUCs of PROS1 were 0.967, 1.000, 0.852, and 1.000 in GSE57555, GSE61850, GSE103909 and TCGA, respectively

When comparing different clinical stages of ICC, hsa-miR-27a-3p was a promising biomarker with an AUC of 0.821. However, the AUC of RP11-328K4.1 was 0.670, and the AUC of PROS1 was 0.554, which suggested that they have limited diagnostic utility (Figure 14D).

## DISCUSSION

ICC, the second most common malignant hepatic tumor, second only to hepatocellular carcinoma (HCC). Although ICC is far less common than extra-cholangiocarcinoma (ECC), the morbidity and mortality rates of ICC have been increasing for the last 10 to 20 years [3, 32, 33]. Therefore, increasing attention has been paid to the pathogenesis and prognosis of ICC [34]. In recent years, the roles of ncRNAs in the pathogenesis and progression of tumors have become increasingly important. The ceRNA hypothesis, which was proposed in 2011, is considered to be a landmark in understanding the mutual regulatory relationship and interactions of RNA-RNA in their entirety. Accumulated evidence suggests that the dysregulation of ceRNA interactions and ceRNETs is involved in the pathogenesis, progression and prognosis of a variety of cancers, including HCC [31] and CCA [29, 35]. Of note, ICC is not only significantly different from HCC in terms of etiology, pathogenesis and invasion and metastasis mode but is also significantly distinct from pCCA and dCCA (another two types of CCA) in terms of anatomical location, pathogenesis and prognosis. Therefore, it is of great importance to further use the ceRNET theory to study the pathogenesis of ICC, to explore the key regulatory pathways causing ICC and to screen potential molecular biomarkers for optimizing individualized and precise therapy of ICC.

Based on the ceRNET theory and the GEO microarray database, for the first time, we constructed ICC-related ceRNETs by using a bioinformatics method, subsequently screened the core regulatory pathway related to the pathogenesis of ICC:RP11-328K4.1-hsa-miR-27a-3p-PROS1, and finally conducted preliminary experimental validation of the expression levels, expression trends and regulatory relationships of the screened ceRNAs of this core regulatory pathway by using molecular experiments. In our previous bioinformatic analysis, the expression of lncRNA RP11-328K4.1 and PROS1 mRNA was downregulated in cancer tissues compared to that in adjacent normal tissues in ICC, while the expression of miRNA hsa-miR-27a-3p was upregulated in ICC cancer tissues, which was consistent with the mechanism of action and expression trends between ceRNA and miRNA in the ceRNA hypothesis. Our subsequent qRT-PCR validation in tissue and plasma also revealed low expression of lncRNA RP11-328K4.1 and PROS1 mRNA but high expression of miRNA hsa-miR-27a-3p in cancer tissue and peripheral plasma compared to the levels observed in adjacent normal tissue and healthy human peripheral plasma. These results suggest that the upregulation of lncRNA RP11-328K4.1 could eliminate the inhibited expression of PROS1 mRNA by oncogenic miRNA hsa-miR-27a through sponge adsorption. Therefore, lncRNA RP11-328K4.1 could exert its role as a tumor suppressor gene in ICC. Meanwhile, the expression of the protein corresponding to PROS1 mRNA was preliminarily validated in the fresh tissue specimens, peripheral plasma and paraffin specimens of ICC patients. In addition, to clinically validate the differential diagnostic ability of the RNAs in this core regulatory pathway of the ICC-related ceRNET, not only in distinguishing ICC tumor tissues and matched adjacent nontumor tissues but also in identifying ICC of different stages, ROC analysis was performed by utilizing data from the NCBI GEO database and the TCGA database. ROC analysis revealed that elements of the core regulatory pathway, including RP11-328K4.1, hsa-miR-27a-3p, and PROS1, might play important roles in ICC diagnosis. Specifically, hsa-miR-27a-3p might have significant diagnostic value in identifying ICC of different clinical stages, while RP11-328K4.1 and PROS1 does not.

The ceRNET constructed in this study contains 340 lncRNA-miRNA-mRNA regulatory relationships. Functional enrichment analysis of 40 DE mRNAs in this regulatory network revealed that this regulatory network is primarily associated with the regulation of proliferation of epithelial cells and activated T cells, which might play a role via the complement and coagulation cascade pathways in ICC. ICC is a malignant tumor derived from the bile duct epithelium at the proximal end of the secondary branch of the intrahepatic bile duct [36]. The proliferation of biliary epithelial cells promotes CCA progression. Studies have shown [37] that the proliferation capacity of activated T and T lymphocyte-mediated killing activity are significantly decreased in patients with malignant tumors. It is conceived that the occurrence of tumors can activate the antitumor immune response of activated T lymphocytes. However, tumor cells and their metabolites will block host T lymphocytes in response to tumor progression, thereby leading to dysregulated metabolism of T lymphocytes, weakened immune response against tumor antigens, hindered differentiation of effector cells and suppressed tumor immunity. In addition, their study indicates that the active proliferative response of T lymphocytes stimulated by antigens is an important part of mediating the effects and roles of cellular immunity *in vivo*. Meanwhile, bioinformatics analysis of proteomics and genomics regarding differentially expressed genes in prostate cancer also shows [38] that the complement and coagulation cascades are involved in the pathogenesis of prostate cancer. However, the specific mechanism of its action in cancer has not yet been reported, but deserves continuous attention and in-depth discussion in the future.

At present, there are few studies on the expression level, diagnostic and prognostic value of lncRNA RP11-328K4.1 in malignant tumors. In a Chinese study on the expression of lncRNA in gastric cancer and its prognostic value by the Center for Gastric Cancer Diagnosis and Treatment of Sun Yat-sen University, Chen W et al. [39] investigated the expression and prognostic value of lncRNAs in gastric cancer tissues. In this study, the expression of lncRNA RP11-328K4.1 was downregulated in gastric cancer tissues compared to normal tissues, which was consistent with the bioinformatic analysis and experimental validation in our study. In addition, their subsequent survival analysis showed that lncRNA RP11-328K4.1 was a protective factor for the prognosis of gastric cancer patients; gastric cancer patients with high expression of lncRNA RP11-328K4.1 had a better prognosis. Therefore, they suggested that lncRNA RP11-328K4.1 is expected to become a new targeted therapeutic target and prognostic molecular marker for gastric cancer. Similarly, in the present study, we found that the expression of lncRNA RP11-328K4.1 was decreased in the cancer tissue and peripheral plasma of ICC patients, suggesting that it may be a protective factor for the prognosis of ICC patients that plays a role as a tumor-suppressor gene. However, the mechanism of how lncRNA RP11-328K4.1 acts as a tumor suppressor gene in gastric cancer is not mentioned in the study by Chen W et al. [39]. In our study, the RP11-328K4.1-hsa-miR-27a-3p-PROS1 regulatory pathway in ICC was detected and can provide ideas and references for the mechanism of action of lncRNA RP11-328K4.1 in gastric cancer.

MicroRNA-27a (miR-27a) is located on chromosome 19. The abnormal expression of miR-27-3p, one of two isoforms of mature miR-27a (the other isoform is miR-27-5p), has been found to play a role in various types of tumors. Wu XZ et al. [40] showed that the expression of miR-27a-3p was significantly increased in esophageal squamous cell carcinoma (ESCC) tissues and cell lines. In Eca109 esophageal cancer cells, ectopic overexpression of miR-27a-3p promoted cell proliferation, while inhibition of miR-27a-3p decreased cell proliferation. Further studies indicated that downregulation of miR-27a-3p expression could induce cell cycle arrest at G1/S. Mechanistic studies demonstrated that miR-27a-3p significantly inhibited the expression of FBXW7. FBXW7 is a tumor suppressor factor that exerts a significant inhibitory effect on the proliferation of Eca109 cells. Finally, they concluded that miR-27a-3p exerted a tumorigenic effect by targeting FBXW7. The above findings suggest that miR-27a-3p may be a potential therapeutic target for ESCC. In addition, Zhou L et al [41] showed that miR-27a-3p was abnormally highly expressed in gastric cancer tissues and cell lines, and this could induce cell cycle arrest at G1/S to initiate the miR-27a-3p/BTG2/Ras/MEK/ERK pathway by inducing B-cell translocation gene 2 (BTG2) (pro-apoptotic), thereby promoting gastric cancer cell proliferation and tumor growth *in vivo* and *in vitro*. The miR-27a-3p/BTG2 axis is believed to be promising as not only a diagnostic biomarker for patients with gastric cancer but also a potential therapeutic target for gastric cancer. Liang J et al. [42] showed that the expression of miR-27a-3p was upregulated in colorectal cancer (CRC) and this was closely associated with histological differentiation, clinical stage, distant metastasis, and survival of CRC patients. Mechanistic studies have shown that miR-27a-3p inhibits apoptosis *in vivo* and *in vitro* and promotes proliferation, migration, and invasion of CRC cells by activating the Wnt/β-catenin pathway via targeting the downstream gene RXRα. Therefore, miR-27a-3p is considered to be a prognostic biomarker and/or a potential therapeutic target for CRC patients. Li L et al. [43] also confirmed that miR-27a-3p was upregulated in nasopharyngeal carcinoma. Mechanistic studies have shown that miR-27a-3p promotes the proliferation, migration, and invasion of nasopharyngeal carcinoma cells by directly inhibiting the 3’ untranslated region (3’-UTR) of Mapk103. Wang WS et al. [44] confirmed that the expression of miR-27a-3p was significantly increased in the peripheral blood of patients with pancreatic cancer and that its expression level can effectively distinguish between pancreatic cancer, benign pancreatic disease, and healthy subjects. Our bioinformatic analysis and experimental validation of miR-27a-3p in ICC are consistent with the above results, confirming the high expression of miR-27a-3p in the cancer tissues and peripheral blood of ICC patients, suggesting that miR-27a-3p might become a potential prognostic and prognostic biomarker and therapeutic target for ICC patients. The mechanism and pattern of miR-27a-3p expression in promoting carcinogenesis in the above-described tumors are similar to the findings in this study on ICC; all of the observed effects depend on the action of miR-27a-3p on downstream mRNA to cause corresponding pathway changes. In turn, these data support the scientific nature of our approach to studying the pathogenesis of ICC by constructing ceRNETs. However, Zhao N et al. [45] suggested that the expression miR-27a-3p is downregulated in cancer tissues and cell lines of HCC, which is significantly associated with early metastasis of HCC. Mechanistic studies have shown that the elevated expression of miR-27a-3p can inhibit metastasis and angiogenesis by directly targeting a vasculogenic mimicry-associated cadherin (VE-cadherin), thereby acting as a tumor suppressor gene. This is inconsistent with the expression trend and role of miR-27a-3p in other types of tumors and our findings in ICC, indicating the universality of tumor heterogeneity and different mechanisms of pathogenesis and biological behavior in different malignancies. The inconsistent expression level and role of miR-27a-3p in HCC and ICC cancer tissues make it useful in clinical practice of hepatic surgery. This is because the early clinical manifestations, tumor markers and imaging signs between HCC and ICC are not distinct, causing great difficulty in differentiating between ICC and HCC during diagnosis, which has been troublesome for hepatic surgeons for a long time. The comparison of the above results suggests that, through further basic experiments and clinical validation, miR-27a-3p is a potentially valuable biomarker for the early distinction of HCC and ICC during diagnosis in the future.

At present, there is a relative lack of studies on the relationship between PROS1 and cancer. Most of the studies were limited to the experimental research level, lacking validation of its clinical diagnostic and prognostic capacities. Che Mat M et al. [46] showed that the expression of PROS1 was significantly increased in pleomorphic glioblastoma and that silencing the expression of PROS1 could effectively reduce the activity of pleomorphic malignant glioblastoma cells, inhibiting their proliferation, migration, and invasion and inducing apoptosis. The same phenomenon was also present in castrated prostate cancer cells. Saraon P et al. [47] showed that the addition of the purified human PROS1 gene significantly increased the migration capacity of these cells, which is inconsistent with our findings. In our study, we show that PROS1 mRNA is significantly decreased in cancer tissues and peripheral plasma of ICC patients, which might play a role as a tumor suppressor gene in ICC. The PROS1 gene encodes a vitamin K-dependent plasma protein, protein S (PS), which is an essential anticoagulant and a multifunctional protein. A lack of PS can cause anticoagulant mechanism disorder, leading to the formation of thrombosis [48]. In the early stage of multiple types of malignant tumors, coagulation dysfunction is first manifested as a hypercoagulable state of the systemic blood that is conducive to the formation of a thrombus. The hypercoagulable state of the blood and the long-term existence of a thrombus further aggravate the progression of malignant tumors [48]. We could boldly speculate that elevated expression of oncogenic miR-27a-3p not only promotes carcinogenesis but also inhibits the expression of PROS1 in ICC patients, thereby supporting the hypercoagulable state of blood and likelihood of thrombosis in ICC patients. Existence of a coagulation disorder and formation of a thrombus, in turn, promote ICC progression. This is also consistent with our pathway enrichment analysis, which shoes that the DE mRNA in ICC are predominantly enriched in the complement and coagulation cascades. As we have also mentioned before, previous studies suggest that complement and coagulation cascades are also involved in the pathogenesis of prostate cancer. However, further basic experiments and clinical studies are still needed regarding the expression level and the specific mechanism of PROS1 mRNA in ICC.

The protein corresponding to PROS1 mRNA had lower overall expression in cancer tissues than in adjacent tissues from the fresh tissue specimens of ICC patients, and this was consistent with the expression trend of PROS1 mRNA in ICC cancer tissues and adjacent tissues, however, there was no statistically significant difference. To our confusion, the protein corresponding to PROS1 mRNA had higher expression in peripheral blood and paraffin sections than in healthy subjects and adjacent tissues; however, this difference was not statistically significant. This reflects the inconsistent expression trend of PROS1 between the mRNA and protein levels in ICC patients. In previous studies, Guoan Chen et al. [49] also observed the same phenomenon in lung adenocarcinoma. They quantitatively analyzed the mRNA and protein expression of 98 genes in lung adenocarcinoma patients. As a result, the expression of only 21 genes was significantly associated with the abundance of its protein; for five of these genes, the protein abundance was significantly different between stage I and stage III lung adenocarcinoma. This suggests a more complex regulatory mechanism between mRNA expression and protein translation. In addition, this may occur for the following reasons: First, the different sensitivities of the detection methods might be responsible. PCR has high specificity for RNA detection, while Western blotting has relatively poor specificity for protein detection. The expression level of mRNA and the level of translated protein are not related in a linear, parallel manner. In addition, posttranslational regulation and modification of proteins transcribed from mRNA lead to different isomers. In this case, increased protein expression level could be observed with Western blotting. Second, protein expression occurs more slowly than mRNA expression. When the amount of mRNA expression decreases, the protein expression could be increased by stabilizing mRNA by increasing the length of the polyA tail (increasing the activity of polyA polymerase) or by decreasing mRNA degradation, causing enhanced mRNA half-life. Third, when mRNA expression is reduced, protein expression could be increased by modifying the protein, decreasing the protein degradation (protease degradation systems such as ubiquitin) and prolonging the half-life of the protein. Fourth, in this study, we detected the mRNA expression in the fresh cancer tissue samples and peripheral plasma of ICC patients by qRT-PCR, and we detected the protein expression in fresh ICC tissue samples, paraffin sections and peripheral plasma from healthy subjects by Western blotting. However, the samples were not completely matched. We can eliminate the effect of this incomplete matching by further expanding the sample size to detect the protein expression in the fresh cancer tissue samples of ICC patients by Western blotting. The detection of protein itself is an indirect method to validate the expression of mRNA. The expression of mRNAs and ncRNAs from the whole core regulatory pathway could be directly detected in the fresh ICC tissue samples by gene sequencing to obtain more direct and reliable outcomes in the future.

There are certain limitations to our study. First, the ceRNETs we constructed are based on the bioinformatic analysis of the expression profile data in the GEO microarray database, while the ICC datasets in the GEO database lack the clinical survival information of patients, leading to the inability to perform prognostic analysis and construct prognostic models and nomograms for the DE ncRNAs (lncRNAs and miRNAs) and DE mRNAs screened in the ceRNA networks. Likewise, the rigor of diagnostic analysis might be limited by the various baseline data of different databases and the small size of the ICC dataset in TCGA. Second, due to the rarity of ICC, the number of ICC specimens used to validate the identified core ceRNA regulatory pathways was relatively small. Although the results of the experimental validation and bioinformatic analysis were consistent, it affected the statistical validity of the experimental validation data to some extent. Third, due to the inconsistent expression trend between experimentally validated mRNA and corresponding protein in different samples, we were unable to perform subsequent prognosis analysis by using IHC analysis of the paraffin specimens of 88 ICC patients, which influenced the detection of prognostic ability of mRNAs in our core regulatory pathway. Fourth, the statistical validity of our ROC analysis, which was conducted to validate the abilities of the RNAs in the core ceRNET regulatory pathway to distinguish ICC tumor tissues and matched adjacent nontumor tissues and to distinguish different tumor stages, was also limited by the sample sizes of datasets in the GEO and TCGA. This suggests that a prospective study should be conducted in the future by performing qRT-PCR or high-throughput sequencing of a large number of fresh tissue samples from ICC patients to enhance the persuasiveness and reliability of the validation and to conduct subsequent prognostic studies.

In conclusion, construction of a ceRNET is an effective way to study the pathogenesis of ICC. The pathogenesis of ICC is associated with the regulation of epithelial cell and activated T lymphocyte proliferation, which also involves the complement and coagulation cascades. Upregulated expression of lncRNA RP11-328K4.1 can eliminate the suppression of PROS1 mRNA expression caused by oncogenic miRNA hsa- miR-27a through sponge adsorption, and this thereby illustrates the protective effect of lncRNA RP11-328K4.1 in ICC inhibition. The lncRNA RP11-328K4.1, miRNA hsa-miR-27a-3p and mRNA PROS1, which are central nodes with high connectivity in the ICC ceRNET, exhibited great ability to distinguish ICC tumor tissues and matched adjacent nontumor tissues and to distinguish ICC tumor stages. They would be ideal diagnostic and prognostic biomarkers and therapeutic targets for ICC patients in the future.

## MATERIALS AND METHODS

### Study flow

**Figure 15:**
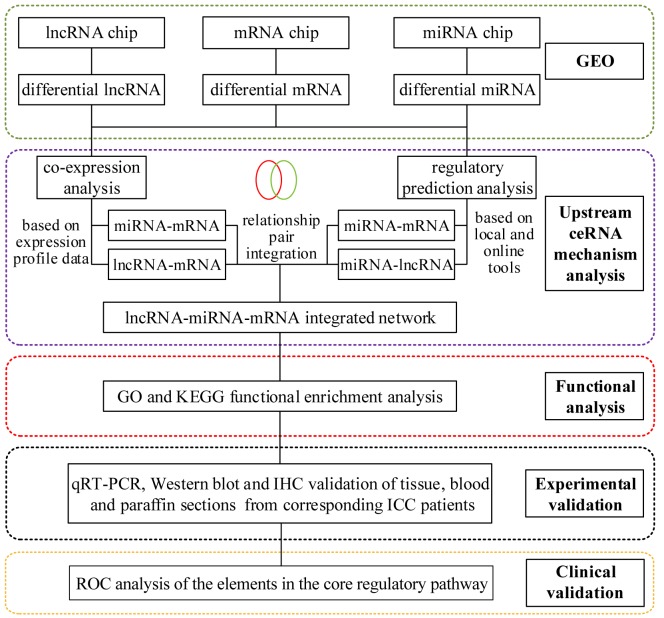
**The flowchart of the study.**

The study was divided into two sections: 1) prediction, construction, bioinformatics screening and functional analysis, and 2) experimental validation. The specific process is shown in Figure 15.

### Analysis of differentially expressed miRNAs, mRNAs and lncRNAs in ICC based on the GEO database

### GEO dataset selection

We searched ICC-related expression profile datasets of lncRNAs, miRNAs and mRNAs in the NCBI GEO (www.ncbi.nlm.nih.gov/geo) database [50]. Only original experimental microarray studies analyzing the expression profile data of lncRNAs, miRNAs, and mRNAs between human ICC or CCA tumor tissue containing ICC and matched nontumor tissue were included. The lncRNA expression profile data were obtained from GSE61850 (five pairs of ICC tumor tissue and matched adjacent nontumor tissue) and GSE103909 (nine pairs of CCA tumor including ICC and adjacent nontumor tissue sample) datasets. miRNA expression profiling data were obtained from GSE53992 (14 pairs of ICC tumor tissues and matched adjacent nontumor tissues), GSE53870 (63 ICC tumor tissues and nine matched adjacent nontumor tissues) and GSE57555 (11 pairs of ICC tumor tissues and matched adjacent nontumor tissues) datasets. mRNA expression profile data were obtained from GSE61850 (five pairs of ICC tumor tissue and matched adjacent nontumor tissue), GSE103909 (nine pairs of CCA tumor including ICC and matched adjacent nontumor tissues) and GSE57555 (11 pairs of ICC tumor tissue and matched adjacent nontumor tissues) datasets.

### Differential expression analysis of ICC-related lncRNAs, miRNAs, and mRNAs in GEO datasets

Differential analysis was performed on lncRNAs, miRNAs and mRNAs in tumor (T), paracancerous © vs normal (N) samples from all datasets by using the classic Bayes method provided by the limma package [51] (Version 3.10.3, http://www.bioconductor.org/packages/2.9/bioc/html/limma.html). Specifically, paired t-tests were used for GSE61850, GSE103909, GSE57555 and GSE53992, while group t-tests were utilized for GSE53870 for significant analysis. All genes were tested to obtain the corresponding P values, followed by multiple test corrections using the Benjamini and Hochberg [52] method to acquire the corrected p-value (adj.P.Value) Log-fold. change (logFC) and significance (p.value or adj.P.Value) were used to measure the differential expression levels of genes. The thresholds were set as follows: GSE61850, mRNA and lncRNA adj.P.Value<0.05 and |logFC|>1; GSE103909, mRNA and lncRNA P.Value<0.05 and |logFC|>0.585; GSE57555, mRNA adj.P.Value< 0.05 and |logFC|>1 and miRNA P. Value<0.05 and |logFC|>0.263; GSE53992, miRNA P.Value<0.05; GSE53870, miRNA adj.P.Value<0.05 and |logFC|>0.585. The differentially expressed (DE) RNA obtained from each dataset are shown as heat maps and volcano maps.

### Data integration

The intersection of DE mRNAs, DE lncRNAs, and DE miRNAs obtained from each dataset is shown in the Venn diagram (http://bioinformatics.psb.ugent.be/Webtools/Venn/).

### Construction of the ceRNA network of ICC based on the GEO database

### Coexpression analysis

We calculated the Pearson correlation coefficient of differentially expressed lncRNAs and differentially expressed mRNA from the GSE61850 and GSE103909 datasets by the one-to-one correspondence of the samples, followed by correlation validation. According to the consistent trend of the expression levels between ceRNAs, lncRNA-mRNA relationship pairs with a Pearson correlation coefficients r>0.7 and a p.values <0.05 were screened, and the two datasets were overlapped. For GSE57555, the Pearson correlation coefficient of differentially expressed mRNA and differentially expressed miRNA was calculated by one-to-one correspondence of the samples, followed by a correlation test. According to the mechanism of miRNA action, that is, blocking the expression of the target gene, miRNA-mRNA relationship pairs with Pearson correlation coefficients less than 0 were screened.

### miRNA target gene prediction analysis

Target gene prediction was performed on miRNAs obtained by differential expression analysis using the online tool mirwalk2.0 (http://zmf.umm.uni-heidelberg.de/apps/zmf/mirwalk2/) [53]. There were 12 databases of miRNA-target gene relationship pairs in miRWalk, including miRWalk, MicroT4, miRanda, miRBridge, miRDB, miRMap, miRNAMap, PICTAR2, PITA, RNA22, RNAhybrid, and Targetscan. The criterion was set as follows: the predicted miRNA-target gene relationship pairs should be present in at least six of the above-described databases, indicating that the miRNA regulates the corresponding target gene. These miRNAs were further intersected with miRNA-mRNA pairs obtained from the above-described coexpression analysis to obtain the final miRNA-mRNA relationship pair.

The DElncRNAs and DEmiRNAs obtained by differential expression analysis were extracted, followed by a prediction of the miRNA-lncRNA binding site using the local software Miranda (v3.3a) [54]. The threshold was set at score>140 and energy<-20, to obtain the final miRNA-lncRNA relationship pairs.

### ceRNA relationship integration and network construction

Based on the miRNA-lncRNA and miRNA-mRNA relationship pair obtained in the previous step, according to the premise that RNAs with the same miRNA binding sites were complementary ceRNAs in the ceRNA network, we first screened the mRNAs and lncRNAs regulated by the same miRNA. Then, according to the consistent trend of expression level of the ceRNAs, combined with the synergistic expression relationship between the mRNA and lncRNA, we obtained the final lncRNA-miRNA-mRNA regulatory relationship. The ceRNA network was constructed using Cytoscape (version 3.4.0, http://chianti.ucsd.edu/cytoscape-3.4.0/) [55] to determine the lncRNA-miRNA-mRNA regulatory relationship obtained above and the expression changes in these nodes. If the upregulation and downregulation patterns of a lncRNA in GSE61850 and GSE103909 were inconsistent, then the lncRNA was not considered to have a regulatory role. If the upregulation and downregulation patterns of a mRNA were not consistent in GSE61850, GSE103909, and GSE57555, then the mRNA was not considered to have a regulatory role. If the upregulation and downregulation of patterns a miRNA in GSE57555, GSE53992, and GSE53870 were not consistent, then the miRNA was not regarded to have a regulatory role. The Cytoscape plugin CytoNCA (Version 2.1.6, http://apps.cytoscape.org/apps/cytonca) [56] was used to analyze the node connectivity of the network, with the parameters were not weighted. The node size in the figure indicates the degree of connectivity in the network: the larger the node, the higher the connectivity.

### Functional enrichment analysis of differentially expressed mRNAs in the constructed ICC-related ceRNETs

To further understand the potential functions and action mechanisms of differentially expressed mRNAs in constructed ceRNETs of ICC, the Database for Annotation, Visualization, and Integrated Discovery (DAVID) bioinformatics tool (https://david.ncifcrf.gov/) version 6.8 was used. Gene Ontology (GO) and Kyoto Encyclopedia of Genes and Genomes (KEGG) pathway enrichment analyses were performed on 44 differentially expressed mRNAs.

### Determination of the ceRNA core regulatory pathway for experimental validation

According to whether the ranking of the network node of differentially expressed RNA was consistent with the expression trend among the various GEO data sets and according to the logFC and ceRNA relationship and previous outcomes in other types of tumors, we determined the core ceRNA regulatory relationships and pathways for further molecular biological experimental validation.

### Molecular biological experimental validation of ICC-related ceRNA core regulatory pathway

### Sample collection

(1) Fresh ICC tissue (obtained by removal of necrotic tissue) and adjacent normal tissues (normal liver tissue more than 1 cm from tumor) were collected from ICC patients admitted to Peking Union Medical College Hospital of China Academy of Medical Sciences from November 2018 to March 2019, and the samples were conserved at -80 °C. (2) Peripheral venous blood was collected from 10 ICC patients three days before surgery and from ten healthy controls with matched baseline characteristics by using EDTA-coated (ethylene diamine tetraacetic acid) anticoagulant tubes. The upper plasma layer was collected after centrifugation at 3000 rpm/min for 15 min and further stored at -80 °C. (3) A total of 88 paraffin-embedded samples were collected from ICC patients who underwent surgery at Peking Union Medical College Hospital of China Academy of Medical Sciences from 2011 to 2016. The sample collection, storage and subsequent experimental procedures were approved by the Peking Union Medical College Ethics Committee. Written informed consent was signed by all patients and healthy donors.

### Quantitative real-time polymerase chain reaction (qRT-PCR)

Total RNA from ICC tissues, adjacent normal tissues and plasma was extracted by using TRIzol reagent (Invitrogen, Life Technologies). Afterwards, the concentration and purity of total RNA were determined by a NanoDrop instrument, and the degradation of RNA was detected by agarose gel electrophoresis. RNA was reverse transcribed into cDNA by using the SuperScriptTM III Reverse Transcriptase (Invitrogen) Kit according to the manufacturer’s instructions. RP11-328K4.1, PROS, β-actin, hsa-miR-27a-3p and U6 primers were all synthesized by Yingjun Biotechnology Co., Ltd. (primer sequences shown in Table 3). Subsequently, 2X Master Mix, forward and reverse primers for each gene, cDNA and ddH_2_O were added and mixed by using the Arraystar Real-Time PCR Kit according to the manufacturer’s instructions, and this was followed by RT-qPCR performed separately in an AB Applied Biosystems Viia7 DX (Life Technologies, USA) (for RP11-328K4.1) or a QuantStudio5 Real-time PCR System (Applied Biosystems) (for hsa-miR-27a-3p and mRNA PROS1). The PCR amplification reaction program was set as the follows: 95 °C, 10 min; 40 PCR cycles (95 °C, 10 sec; 60 °C, 60 sec). After completion of the amplification reaction, a melt curve of the PCR product was established (95 °C, 10 sec; 60 °C, 60 sec; 95 °C, 15 sec). The expression of hsa-miR-27a-3p was normalized to the internal control, U6. The expression of IncRNA RP11-328K4.1 and PROS1 mRNA was normalized to the internal control, β-actin. The 2-ΔΔCT method was used for data analysis.

**Table 3:** PCR primers.

**Gene**	**Primer (5′→3′)**
RP11-328K4.1	F: TTGTTTTGCTTATTGGTGTTTA
	R: CAGAGTCAGTCTCCTCATTTCA
PROS	F: CCGATTAACCCTCGTCTA
	R: CAAGGCAAGCATAACACC
β-actin (H)	F: GTGGCCGAGGACTTTGATTG
	R: CCTGTAACAACGCATCTCATATT
hsa-miR-27a-3p	GSP: GGGTTCACAGTGGCTAAG
	R: CAGTGCGTGTCGTGGA
U6	F: GCTTCGGCAGCACATATACTAAAAT
	R: CGCTTCACGAATTTGCGTGTCAT

F: forward primer, R: reverse primer, GSP: specific primer for corresponding miRNA.

### Western blot analysis

Total protein from ICC tissues and adjacent noncancer tissues was extracted with protein extraction reagent (KangChen, KC-415) containing protease inhibitor, PMSF and phosphatase inhibitor, followed by protein quantification by using the BCA Protein Quantitation Kit (Kang Chen, KC-430) according to the manufacturer’s instructions. Protein samples were separated by 10% SDS-PAGE and transferred to PVDF (Polyvinylidene Difluoride) membranes (Millipore, Bedford, MA, USA) at a constant current of 120 mA. The PVDF membrane was blocked with 5% BSA on a shaker at room temperature for 1 h. Afterwards, the membrane was incubated with anti-PROS1 antibody (1:1000 dilution, Proteintech) or anti-β-actin antibody (1:5000 dilution, Kangcheng) at 4 °C overnight. After washing with TBST, the membrane was reacted with the appropriate anti-IgG antibody (1:5000 dilution, Shanghai Kangcheng Bioengineering Co., Ltd.) on a shaker at room temperature for 1 h. After washing with TBST, KC^TM^ chemiluminescence solution (KangChen, KC-420) was added dropwise to the membrane, followed by visualization of the protein band with X-ray film in the dark. ImageJ software was used to determine the grayscale value of the protein band.

### Immunohistochemistry (IHC)

Paraffin-embedded ICC tissue samples were sectioned into 5 μm-thick tissue sections. Each slice was hydrated with an ethanol gradient and then placed in PBS buffer under high pressure for antigen retrieval. Endogenous peroxidase was blocked with 3% hydrogen peroxide. Then, rabbit anti-PROS1 polyclonal antibody (Proteintech, 1:20-1:200, AG10539) was added dropwise to the slices and incubated for 90 min at room temperature. After rinsing with PBS buffer, HRP-labeled goat anti-rabbit IgG antibody (KPL, 1:200, 074-1506) was added dropwise and incubated at room temperature for 30 min. After rinsing, diaminobenzidine substrate solution (DAB kit) was added to the slices for visualization. Specific staining of tissue sections was observed under a microscope (Nikon), and the number of positive cells was counted from at least five views of each region.

### Clinical validation of the ICC-related ceRNA core regulatory pathway

Expression profiles of RP11-328K4.1, hsa-miR-27a-3p, and PROS1 in ICC tumor tissues and matched adjacent nontumor tissues were obtained from the TCGA database and the NCBI GEO database. The pROC package [57] was used to plot ROC curves and to calculate the area under the ROC curve (AUC). To assess the diagnostic capacity of the elements of the core regulatory pathways in different stages of ICC, expression profiles of T1 and T2 stage in TCGA were merged into the early group, and those of T3 stage were the advanced group for ROC analysis.

### Statistical analysis

Statistical analysis was performed using SPSS software. Data with a normal distribution are shown as the mean ± standard deviation, and data without a normal distribution are shown as the median and interquartile range. An independent sample t-test was used to compare two groups. A P < 0.05 was considered statistically significant.

## Supplementary Material

Supplementary Figures

Supplementary Tables

## References

[1] Fitzmaurice C, Dicker D, Pain A, Hamavid H, Moradi-Lakeh M, MacIntyre MF, Allen C, Hansen G, Woodbrook R, Wolfe C, Hamadeh RR, Moore A, Werdecker A, et al, and Global Burden of Disease Cancer Collaboration. The Global Burden of Cancer 2013. JAMA Oncol. 2015; 1:505–27. 10.1001/jamaoncol.2015.0735 26181261 PMC4500822

[2] Aljiffry M, Abdulelah A, Walsh M, Peltekian K, Alwayn I, Molinari M. Evidence-based approach to cholangiocarcinoma: a systematic review of the current literature. J Am Coll Surg. 2009; 208:134–47. 10.1016/j.jamcollsurg.2008.09.007 19228515

[3] Shaib YH, Davila JA, McGlynn K, El-Serag HB. Rising incidence of intrahepatic cholangiocarcinoma in the United States: a true increase? J Hepatol. 2004; 40:472–77. 10.1016/j.jhep.2003.11.030 15123362

[4] Bridgewater J, Galle PR, Khan SA, Llovet JM, Park JW, Patel T, Pawlik TM, Gores GJ. Guidelines for the diagnosis and management of intrahepatic cholangiocarcinoma. J Hepatol. 2014; 60:1268–89. 10.1016/j.jhep.2014.01.021 24681130

[5] Weber SM, Ribero D, O’Reilly EM, Kokudo N, Miyazaki M, Pawlik TM. Intrahepatic cholangiocarcinoma: expert consensus statement. HPB (Oxford). 2015; 17:669–80. 10.1111/hpb.12441 26172134 PMC4527852

[6] Dodson RM, Weiss MJ, Cosgrove D, Herman JM, Kamel I, Anders R, Geschwind JF, Pawlik TM. Intrahepatic cholangiocarcinoma: management options and emerging therapies. J Am Coll Surg. 2013; 217:736–750.e4. 10.1016/j.jamcollsurg.2013.05.021 23890842

[7] Kataoka M, Wang DZ. Non-Coding RNAs Including miRNAs and lncRNAs in Cardiovascular Biology and Disease. Cells. 2014; 3:883–98. 10.3390/cells3030883 25153164 PMC4197640

[8] Qiu MT, Hu JW, Yin R, Xu L. Long noncoding RNA: an emerging paradigm of cancer research. Tumour Biol. 2013; 34:613–20. 10.1007/s13277-013-0658-6 23359273

[9] Ponting CP, Oliver PL, Reik W. Evolution and functions of long noncoding RNAs. Cell. 2009; 136:629–41. 10.1016/j.cell.2009.02.006 19239885

[10] Hung T, Chang HY. Long noncoding RNA in genome regulation: prospects and mechanisms. RNA Biol. 2010; 7:582–85. 10.4161/rna.7.5.13216 20930520 PMC3073254

[11] Ma SL, Li AJ, Hu ZY, Shang FS, Wu MC. Co-expression of the carbamoyl-phosphate synthase 1 gene and its long non-coding RNA correlates with poor prognosis of patients with intrahepatic cholangiocarcinoma. Mol Med Rep. 2015; 12:7915–26. 10.3892/mmr.2015.4435 26499888 PMC4758274

[12] Wang J, Xie H, Ling Q, Lu D, Lv Z, Zhuang R, Liu Z, Wei X, Zhou L, Xu X, Zheng S. Coding-noncoding gene expression in intrahepatic cholangiocarcinoma. Transl Res. 2016; 168:107–21. 10.1016/j.trsl.2015.07.007 26297049

[13] Greco S, Gorospe M, Martelli F. Noncoding RNA in age-related cardiovascular diseases. J Mol Cell Cardiol. 2015; 83:142–55. 10.1016/j.yjmcc.2015.01.011 25640162 PMC5509469

[14] Volinia S, Galasso M, Costinean S, Tagliavini L, Gamberoni G, Drusco A, Marchesini J, Mascellani N, Sana ME, Abu Jarour R, Desponts C, Teitell M, Baffa R, et al. Reprogramming of miRNA networks in cancer and leukemia. Genome Res. 2010; 20:589–99. 10.1101/gr.098046.109 20439436 PMC2860161

[15] Chen L, Yan HX, Yang W, Hu L, Yu LX, Liu Q, Li L, Huang DD, Ding J, Shen F, Zhou WP, Wu MC, Wang HY. The role of microRNA expression pattern in human intrahepatic cholangiocarcinoma. J Hepatol. 2009; 50:358–69. 10.1016/j.jhep.2008.09.015 19070389

[16] Oishi N, Kumar MR, Roessler S, Ji J, Forgues M, Budhu A, Zhao X, Andersen JB, Ye QH, Jia HL, Qin LX, Yamashita T, Woo HG, et al. Transcriptomic profiling reveals hepatic stem-like gene signatures and interplay of miR-200c and epithelial-mesenchymal transition in intrahepatic cholangiocarcinoma. Hepatology. 2012; 56:1792–803. 10.1002/hep.25890 22707408 PMC3458130

[17] Silakit R, Loilome W, Yongvanit P, Chusorn P, Techasen A, Boonmars T, Khuntikeo N, Chamadol N, Pairojkul C, Namwat N. Circulating miR-192 in liver fluke-associated cholangiocarcinoma patients: a prospective prognostic indicator. J Hepatobiliary Pancreat Sci. 2014; 21:864–72. 10.1002/jhbp.145 25131257

[18] Plieskatt JL, Rinaldi G, Feng Y, Peng J, Yonglitthipagon P, Easley S, Laha T, Pairojkul C, Bhudhisawasdi V, Sripa B, Brindley PJ, Mulvenna JP, Bethony JM. Distinct miRNA signatures associate with subtypes of cholangiocarcinoma from infection with the tumourigenic liver fluke Opisthorchis viverrini. J Hepatol. 2014; 61:850–58. 10.1016/j.jhep.2014.05.035 25017828

[19] Li L, Masica D, Ishida M, Tomuleasa C, Umegaki S, Kalloo AN, Georgiades C, Singh VK, Khashab M, Amateau S, Li Z, Okolo P, Lennon AM, et al. Human bile contains microRNA-laden extracellular vesicles that can be used for cholangiocarcinoma diagnosis. Hepatology. 2014; 60:896–907. 10.1002/hep.27050 24497320 PMC4121391

[20] Tay Y, Kats L, Salmena L, Weiss D, Tan SM, Ala U, Karreth F, Poliseno L, Provero P, Di Cunto F, Lieberman J, Rigoutsos I, Pandolfi PP. Coding-independent regulation of the tumor suppressor PTEN by competing endogenous mRNAs. Cell. 2011; 147:344–57. 10.1016/j.cell.2011.09.029 22000013 PMC3235920

[21] Cesana M, Cacchiarelli D, Legnini I, Santini T, Sthandier O, Chinappi M, Tramontano A, Bozzoni I. A long noncoding RNA controls muscle differentiation by functioning as a competing endogenous RNA. Cell. 2011; 147:358–69. 10.1016/j.cell.2011.09.028 22000014 PMC3234495

[22] Sumazin P, Yang X, Chiu HS, Chung WJ, Iyer A, Llobet-Navas D, Rajbhandari P, Bansal M, Guarnieri P, Silva J, Califano A. An extensive microRNA-mediated network of RNA-RNA interactions regulates established oncogenic pathways in glioblastoma. Cell. 2011; 147:370–81. 10.1016/j.cell.2011.09.041 22000015 PMC3214599

[23] Tay Y, Karreth FA, Pandolfi PP. Aberrant ceRNA activity drives lung cancer. Cell Res. 2014; 24:259–60. 10.1038/cr.2014.21 24525785 PMC3945890

[24] Hu Y, Tian H, Xu J, Fang JY. Roles of competing endogenous RNAs in gastric cancer. Brief Funct Genomics. 2016; 15:266–73. 10.1093/bfgp/elv036 26404556

[25] Xia T, Liao Q, Jiang X, Shao Y, Xiao B, Xi Y, Guo J. Long noncoding RNA associated-competing endogenous RNAs in gastric cancer. Sci Rep. 2014; 4:6088. 10.1038/srep06088 25124853 PMC4133709

[26] Liang WC, Fu WM, Wong CW, Wang Y, Wang WM, Hu GX, Zhang L, Xiao LJ, Wan DC, Zhang JF, Waye MM. The lncRNA H19 promotes epithelial to mesenchymal transition by functioning as miRNA sponges in colorectal cancer. Oncotarget. 2015; 6:22513–25. 10.18632/oncotarget.4154 26068968 PMC4673179

[27] Yang J, Li T, Gao C, Lv X, Liu K, Song H, Xing Y, Xi T. FOXO1 3'UTR functions as a ceRNA in repressing the metastases of breast cancer cells via regulating miRNA activity. FEBS Lett. 2014; 588:3218–24. 10.1016/j.febslet.2014.07.003 25017439

[28] Lin P, Wen DY, Li Q, He Y, Yang H, Chen G. Genome-Wide Analysis of Prognostic lncRNAs, miRNAs, and mRNAs Forming a Competing Endogenous RNA Network in Hepatocellular Carcinoma. Cell Physiol Biochem. 2018; 48:1953–67. 10.1159/000492519 30092571

[29] Song W, Miao DL, Chen L. Comprehensive analysis of long noncoding RNA-associated competing endogenous RNA network in cholangiocarcinoma. Biochem Biophys Res Commun. 2018; 506:1004–12. 10.1016/j.bbrc.2018.10.186 30404735

[30] Cao MR, Han ZP, Liu JM, Li YG, Lv YB, Zhou JB, He JH. Bioinformatic analysis and prediction of the function and regulatory network of long non-coding RNAs in hepatocellular carcinoma. Oncol Lett. 2018; 15:7783–93. 10.3892/ol.2018.8271 29740493 PMC5934726

[31] Yan Y, Yu J, Liu H, Guo S, Zhang Y, Ye Y, Xu L, Ming L. Construction of a long non-coding RNA-associated ceRNA network reveals potential prognostic lncRNA biomarkers in hepatocellular carcinoma. Pathol Res Pract. 2018; 214:2031–38. 10.1016/j.prp.2018.09.022 30316688

[32] Bergquist A, von Seth E. Epidemiology of cholangiocarcinoma. Best Pract Res Clin Gastroenterol. 2015; 29:221–32. 10.1016/j.bpg.2015.02.003 25966423

[33] Patel T. Increasing incidence and mortality of primary intrahepatic cholangiocarcinoma in the United States. Hepatology. 2001; 33:1353–57. 10.1053/jhep.2001.25087 11391522

[34] Sirica AE, Gores GJ, Groopman JD, Selaru FM, Strazzabosco M, Wei Wang X, Zhu AX. Intrahepatic Cholangiocarcinoma: Continuing Challenges and Translational Advances. Hepatology. 2019; 69:1803–15. 10.1002/hep.30289 30251463 PMC6433548

[35] Wan M, Zhang FM, Li ZL, Kang PC, Jiang PM, Wang YM, Wang ZD, Zhong XY, Li CL, Wang H, Zhao SY, Cui YF. Identifying survival-associated ceRNA clusters in cholangiocarcinoma. Oncol Rep. 2016; 36:1542–50. 10.3892/or.2016.4934 27432084

[36] Squires MH, Cloyd JM, Dillhoff M, Schmidt C, Pawlik TM. Challenges of surgical management of intrahepatic cholangiocarcinoma. Expert Rev Gastroenterol Hepatol. 2018; 12:671–81. 10.1080/17474124.2018.1489229 29911912

[37] Hang JM, Xie ZH. Preliminary analysis of the correlation between the proliferative response of PHA-activated T lymphocytes and its mediated killing activity in patients with malignant tumors. Chin J Immunol. 1994:214–17.

[38] Chen C, Cao XG, Zhang LG, Me AL, Liu J, Kang SC, Gao W, Han H, Cao FH, Li ZG. Bioinformatics analysis of differential genes from proteomics and genomics in prostate cancer based on literature mining. Chinese Journal of General Practice. 2015; 18:4011–16.

[39] Chen W, Dan WG, Zhang CH, He YL. The expression and prognostic value of lncRNA in gastric cancer. Journal of Digestive Oncology. 2016; 8:254–61.

[40] Wu XZ, Wang KP, Song HJ, Xia JH, Jiang Y, Wang YL. MiR-27a-3p promotes esophageal cancer cell proliferation via F-box and WD repeat domain-containing 7 (FBXW7) suppression. Int J Clin Exp Med. 2015; 8:15556–62. 26629048 PMC4658937

[41] Zhou L, Liang X, Zhang L, Yang L, Nagao N, Wu H, Liu C, Lin S, Cai G, Liu J. MiR-27a-3p functions as an oncogene in gastric cancer by targeting BTG2. Oncotarget. 2016; 7:51943–54. 10.18632/oncotarget.10460 27409164 PMC5239526

[42] Liang J, Tang J, Shi H, Li H, Zhen T, Duan J, Kang L, Zhang F, Dong Y, Han A. miR-27a-3p targeting RXRα promotes colorectal cancer progression by activating Wnt/β-catenin pathway. Oncotarget. 2017; 8:82991–3008. 10.18632/oncotarget.19635 29137318 PMC5669944

[43] Li L, Luo Z. Dysregulated miR-27a-3p promotes nasopharyngeal carcinoma cell proliferation and migration by targeting Mapk10. Oncol Rep. 2017; 37:2679–87. 10.3892/or.2017.5544 28393229 PMC5428281

[44] Wang WS, Liu LX, Li GP, Chen Y, Li CY, Jin DY, Wang XL. Combined serum CA19-9 and miR-27a-3p in peripheral blood mononuclear cells to diagnose pancreatic cancer. Cancer Prev Res (Phila). 2013; 6:331–38. 10.1158/1940-6207.CAPR-12-0307 23430754

[45] Zhao N, Sun H, Sun B, Zhu D, Zhao X, Wang Y, Gu Q, Dong X, Liu F, Zhang Y, Li X. miR-27a-3p suppresses tumor metastasis and VM by down-regulating VE-cadherin expression and inhibiting EMT: an essential role for Twist-1 in HCC. Sci Rep. 2016; 6:23091. 10.1038/srep23091 26980408 PMC4793289

[46] Che Mat MF, Abdul Murad NA, Ibrahim K, Mohd Mokhtar N, Wan Ngah WZ, Harun R, Jamal R. Silencing of PROS1 induces apoptosis and inhibits migration and invasion of glioblastoma multiforme cells. Int J Oncol. 2016; 49:2359–66. 10.3892/ijo.2016.3755 27840905

[47] Saraon P, Musrap N, Cretu D, Karagiannis GS, Batruch I, Smith C, Drabovich AP, Trudel D, van der Kwast T, Morrissey C, Jarvi KA, Diamandis EP. Proteomic profiling of androgen-independent prostate cancer cell lines reveals a role for protein S during the development of high grade and castration-resistant prostate cancer. J Biol Chem. 2012; 287:34019–31. 10.1074/jbc.M112.384438 22908226 PMC3464512

[48] Schwarz HP, Fischer M, Hopmeier P, Batard MA, Griffin JH. Plasma protein S deficiency in familial thrombotic disease. Blood. 1984; 64:1297–300. 10.1182/blood.V64.6.1297.1297 6238642

[49] Chen G, Gharib TG, Huang CC, Taylor JM, Misek DE, Kardia SL, Giordano TJ, Iannettoni MD, Orringer MB, Hanash SM, Beer DG. Discordant protein and mRNA expression in lung adenocarcinomas. Mol Cell Proteomics. 2002; 1:304–13. 10.1074/mcp.M200008-MCP200 12096112

[50] Edgar R, Domrachev M, Lash AE. Gene Expression Omnibus: NCBI gene expression and hybridization array data repository. Nucleic Acids Res. 2002; 30:207–10. 10.1093/nar/30.1.207 11752295 PMC99122

[51] Ritchie ME, Phipson B, Wu D, Hu Y, Law CW, Shi W, Smyth GK. limma powers differential expression analyses for RNA-sequencing and microarray studies. Nucleic Acids Res. 2015; 43:e47. 10.1093/nar/gkv007 25605792 PMC4402510

[52] Green GH, Diggle PJ. On the operational characteristics of the Benjamini and Hochberg False Discovery Rate procedure. Stat Appl Genet Mol Biol. 2007; 6:Article27. 10.2202/1544-6115.1302 18052910

[53] Dweep H, Gretz N. miRWalk2.0: a comprehensive atlas of microRNA-target interactions. Nat Methods. 2015; 12:697. 10.1038/nmeth.3485 26226356

[54] Enright AJ, John B, Gaul U, Tuschl T, Sander C, Marks DS. MicroRNA targets in Drosophila. Genome Biol. 2003; 5:R1. 10.1186/gb-2003-5-1-r1 14709173 PMC395733

[55] Shannon P, Markiel A, Ozier O, Baliga NS, Wang JT, Ramage D, Amin N, Schwikowski B, Ideker T. Cytoscape: a software environment for integrated models of biomolecular interaction networks. Genome Res. 2003; 13:2498–504. 10.1101/gr.1239303 14597658 PMC403769

[56] Tang Y, Li M, Wang J, Pan Y, Wu FX. CytoNCA: a cytoscape plugin for centrality analysis and evaluation of protein interaction networks. Biosystems. 2015; 127:67–72. 10.1016/j.biosystems.2014.11.005 25451770

[57] Robin X, Turck N, Hainard A, Tiberti N, Lisacek F, Sanchez JC, Müller M. pROC: an open-source package for R and S+ to analyze and compare ROC curves. BMC Bioinformatics. 2011; 12:77. 10.1186/1471-2105-12-77 21414208 PMC3068975

