# Supplementary material for: CeRNA regulatory network-based analysis to study the roles of noncoding RNAs in the pathogenesis of intrahepatic cholangiocellular carcinoma
Source: Aging (Albany NY). 2020 Jan 19;12(2):1047–86. doi: 10.18632/aging.102634 (PMC7053603; doi:10.18632/aging.102634)
Supplement: Supplementary Figures [file aging-12-102634-s002..pdf]

SUPPLEMENTARY FIGURES

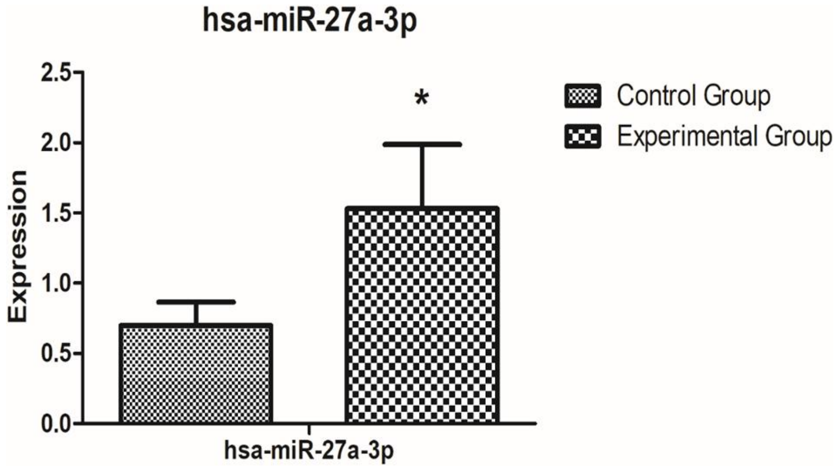

Supplementary Figure 1. qRT-PCR analysis revealed the difference in miRNA hsa-miR-27a-3p expression between the experimental group and control group in fresh ICC tissue samples after normalization to internal controls. hsa-miR-27a-3p was normalized to U6.

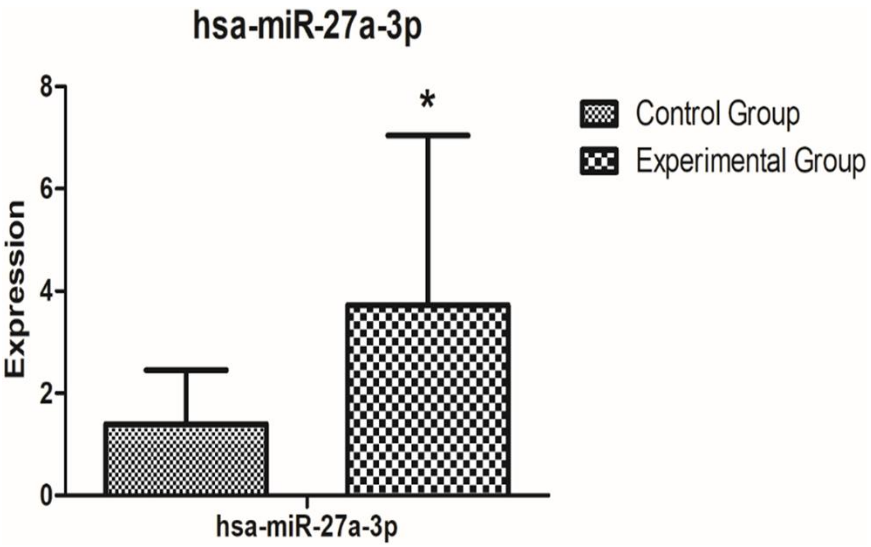

Supplementary Figure 2. qRT-PCR analysis showed the difference in miRNA hsa-miR-27a-3p expression between the experimental group and control group in peripheral plasma samples after normalization to internal controls. hsa-miR-27a-3p was normalized to U6.

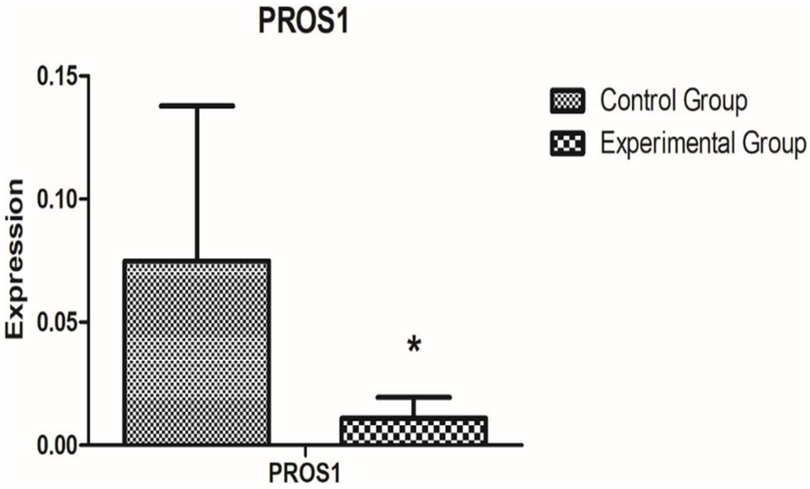

Supplementary Figure 3. qRT-PCR analysis showed the difference in PROS1 mRNA expression between the experimental group and control group in fresh ICC tissue samples after normalization to internal controls. PROS1 was normalized to β-actin.

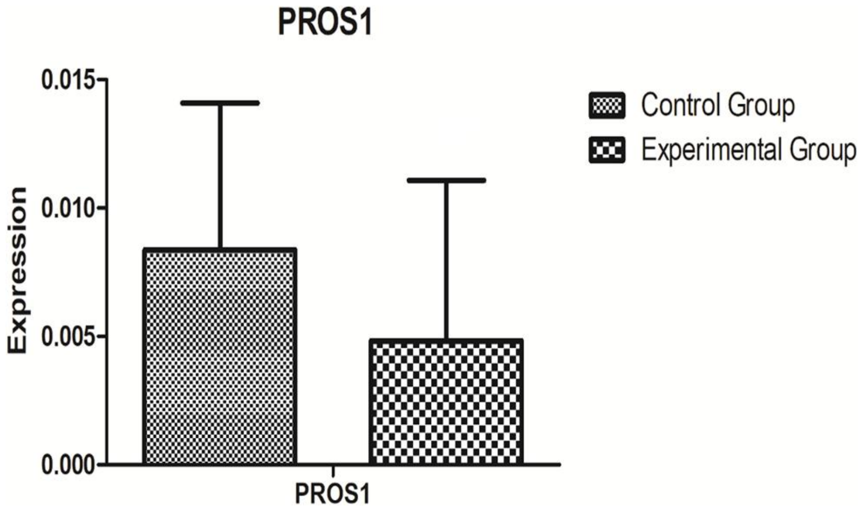

Supplementary Figure 4. qRT-PCR analysis showed the difference in PROS1 mRNA expression between the experimental group and control group in peripheral plasma samples after normalization to internal controls. PROS1 was normalized to β-actin.

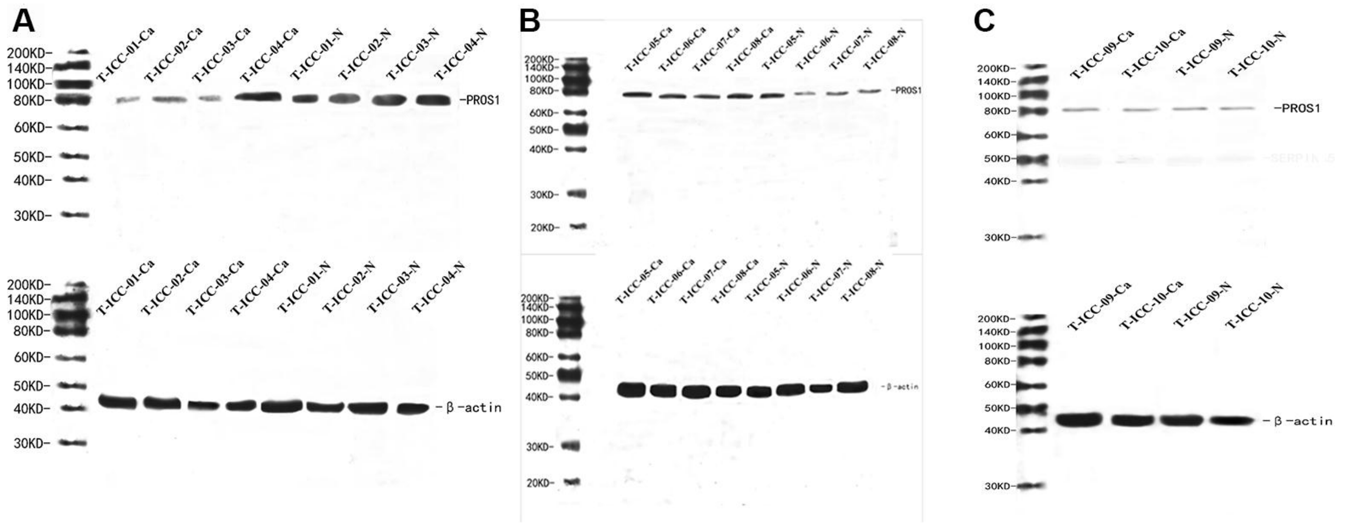

Supplementary Figure 5. (**A**–**C**) Western blot analysis showed the difference in the protein expression of PROS1 between the control group (paracancerous tissue) and the experimental group (ICC cancer tissue) specimens of ICC patients. Ca indicates the experimental group, and N indicates the control group.

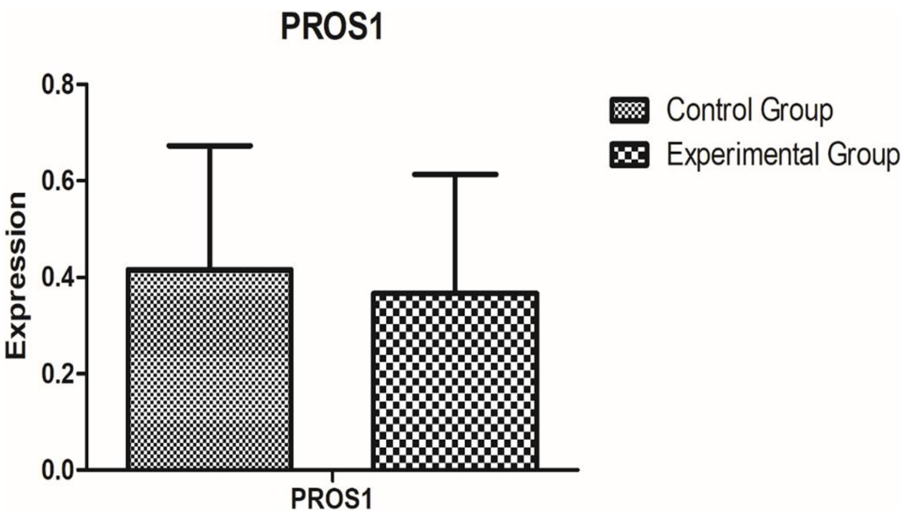

Supplementary Figure 6. The grayscale comparison of the protein expression of PROS1 in the control group (paracancerous tissue) and the experimental group (ICC cancer tissue) of ICC patients detected by Western blotting.

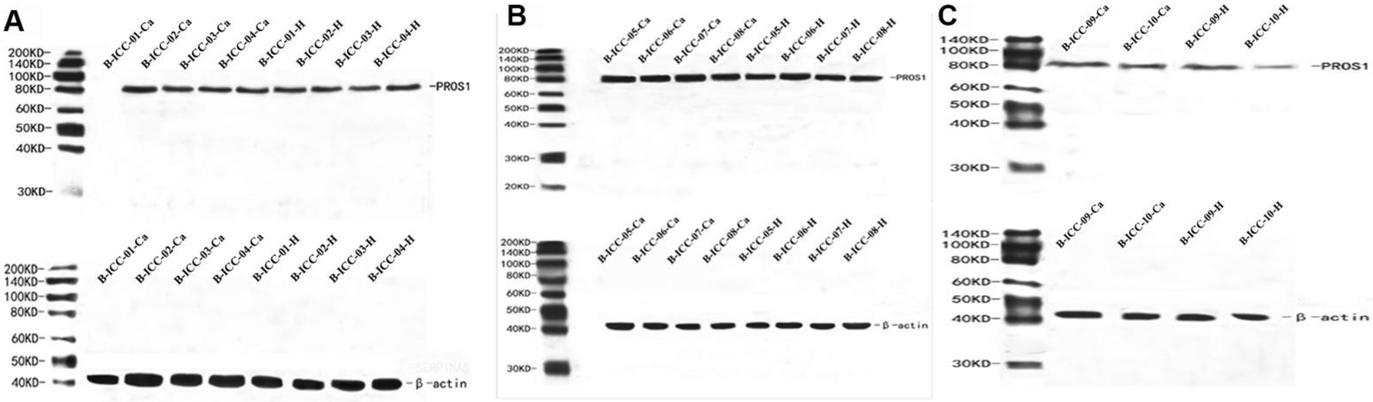

Supplementary Figure 7. (**A**–**C**) Western blot analysis showed the difference in the protein expression of PROS1 in peripheral plasma between the control group and the experimental group. Ca indicates the experimental group, and N indicates the control group.

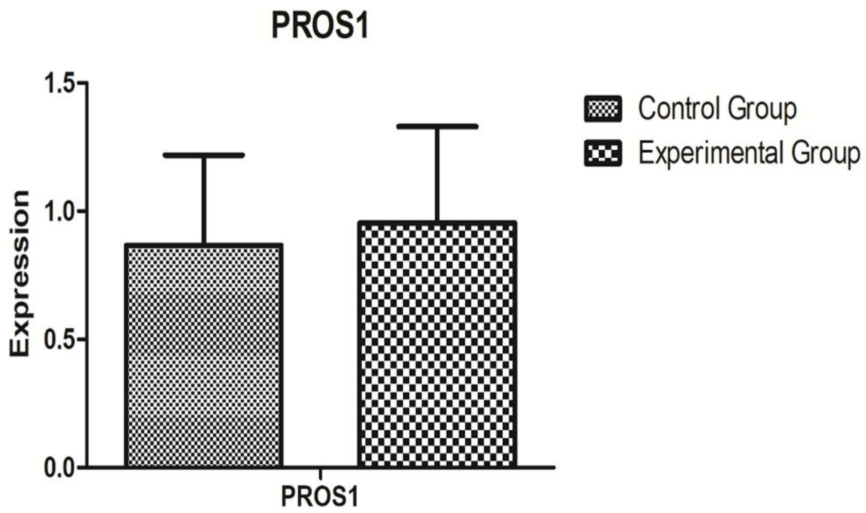

Supplementary Figure 8. The grayscale comparison of the protein expression of PROS1 in peripheral plasma between the control group and the experimental group detected by Western blotting.

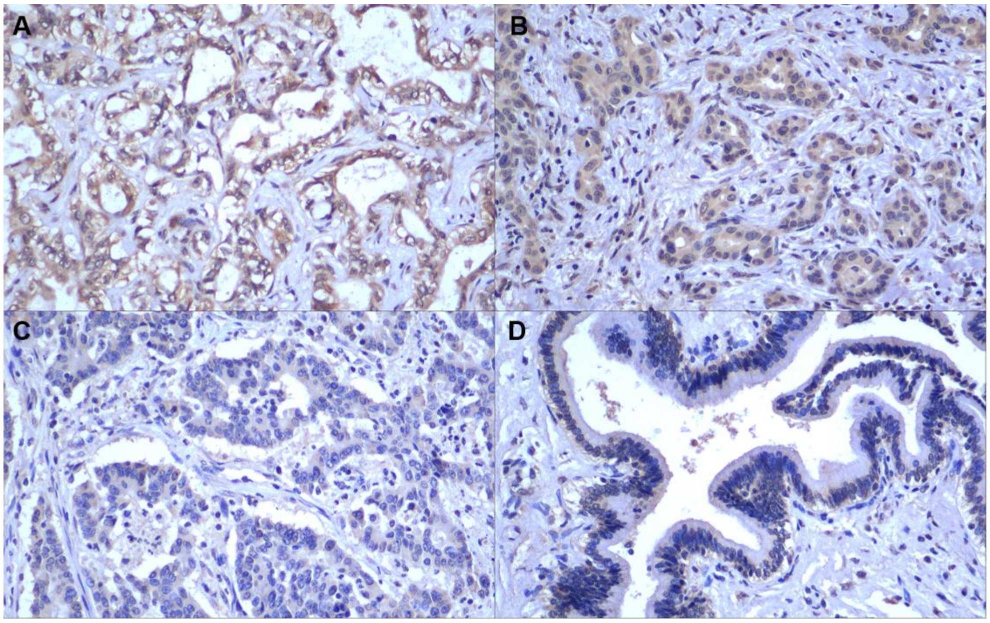

Supplementary Figure 9. (**A**–**D**) The expression of PROS1 in ICC cancer tissues and adjacent normal tissues (both magnified 200 times under a microscope). Figure (**A**–**C**) shows the high, medium and low intensity of PROS1 expression in ICC cancer tissues. (**D**) shows the expression intensity of PROS1 in normal adjacent tissues.
