# Supplementary material for: CeRNA regulatory network-based analysis to study the roles of noncoding RNAs in the pathogenesis of intrahepatic cholangiocellular carcinoma
Source: Aging (Albany NY). 2020 Jan 19;12(2):1047–86. doi: 10.18632/aging.102634 (PMC7053603; doi:10.18632/aging.102634)
Supplement: Supplementary Tables [file aging-12-102634-s001..pdf]

SUPPLEMENTARY TABLES

Supplementary Table 1. Partial data used for the heat map and volcano map, which are shown in Figure 2.

mRNA	logFC	AveExpr	t	P.Value	adj.P.Val	B
SPP2	-7.46113	1.083621	-36.4085	3.66E-09	6.18E-06	10.97354
ASPDH	-7.43768	-0.7132	-33.2134	6.89E-09	6.18E-06	10.58324
DIO1	-7.38381	-1.81958	-19.8824	2.32E-07	3.12E-05	7.859624
NR1I2	-7.36892	-1.30911	-28.5065	1.97E-08	6.53E-06	9.863584
HFE2	-7.0824	-0.56367	-26.7451	3.05E-08	8.20E-06	9.538484
SLC13A5	-7.07661	0.712433	-33.5333	6.45E-09	6.18E-06	10.62548
GDF2	-7.05172	-2.59008	-28.0007	2.23E-08	7.08E-06	9.773759
MOGAT2	-7.03964	-2.36947	-29.7618	1.47E-08	6.36E-06	10.07516
ABCG5	-6.95963	-1.38571	-28.9127	1.79E-08	6.50E-06	9.933778
SLC1A2	-6.95539	0.128473	-31.633	9.64E-09	6.18E-06	10.36303

Supplementary Table 2. Partial data used for the heat map and volcano map, which are shown in Figure 3.

lncRNA	logFC	AveExpr	t	P.Value	adj.P.Val	B
A1BG-AS1	0.65408	-1.98753	2.572355	0.037251	0.10418	-4.6229
A2M-AS1	-0.66045	-0.52705	-3.14625	0.016486	0.062544	-3.77236
AA06	0.980128	-4.54291	2.78679	0.027356	0.085065	-4.30342
AATBC	0.463641	-2.64832	2.159106	0.068152	0.156847	-5.23523
ABALON	0.45691	-3.97925	1.597015	0.154796	0.276406	-6.02634
ABHD11-AS1	0.608142	-4.14432	2.963899	0.021274	0.072385	-4.04072
AC000067.1	1.587322	-6.59809	4.286306	0.003726	0.024653	-2.18003
AC000068.5	0.617275	-2.74619	2.399994	0.047874	0.123122	-4.87953
AC000095.11	0.008573	-5.25044	0.028827	0.977817	0.986028	-7.26132
AC000111.6	1.158307	-6.70436	2.046788	0.080382	0.175714	-5.39883

Supplementary Table 3. Partial data used for the heat map and volcano map, which are shown in Figure 4.

mRNA	logFC	AveExpr	t	P.Value	adj.P.Val	B
PAH	-4.55162	0.009728	-2.50052	0.029807	0.420194	-3.34572
ALB	-4.26961	0.901973	-2.07149	0.063038	0.457798	-3.93243
AHSG	-4.2643	1.348979	-2.01834	0.069048	0.463418	-4.00296
APOH	-4.14661	2.161237	-2.13924	0.056092	0.446171	-3.84166
CRP	-3.99838	1.482156	-2.71715	0.020308	0.407668	-3.04304
SERPINC1	-3.97214	0.727557	-1.84267	0.092931	0.482655	-4.23104
CYP4A11	-3.94755	0.066893	-1.94236	0.078576	0.472544	-4.10262
SULT2A1	-3.8835	0.235051	-1.75875	0.106839	0.492011	-4.33682
HRG	-3.87318	0.898442	-1.81924	0.096637	0.485545	-4.2608
CYP2A7	-3.81305	0.218281	-1.91201	0.082714	0.474909	-4.14202

Supplementary Table 4. Partial data used for the heat map and volcano map, which are shown in Figure 5.

lnc	logFC	AveExpr	t	P.Value	adj.P.Val	B
A1BG-AS1	0.049647	-1.65149	0.24432	0.811556	0.936493	-5.57293
A2M-AS1	-0.45159	-1.43034	-1.44543	0.176682	0.558864	-4.70836
AA06	-0.00068	-1.89126	-0.0015	0.998827	0.999484	-5.60109
AATBC	-0.08132	-1.18167	-0.46454	0.651471	0.870353	-5.50012
ABALON	-0.24596	-1.48391	-1.4461	0.176497	0.558535	-4.70761
ABHD11-AS1	0.789853	-1.93259	2.615783	0.024308	0.411877	-3.1849
AC000067.1	-0.1894	-5.84724	-1.05229	0.315609	0.675434	-5.10558
AC000068.5	-0.12718	-3.31176	-0.84586	0.415951	0.747094	-5.27469
AC000095.11	-0.58473	-3.88572	-3.4773	0.005301	0.40026	-1.99064
AC000111.6	-0.19752	-3.60153	-0.78261	0.450649	0.765889	-5.32023

Supplementary Table 5. Partial data used for the heat map and volcano map, which are shown in Figure 6.

mRNA	logFC	AveExpr	t	P.Value	adj.P.Val	B
SYNGR2	1.952891	9.695844	8.707606	6.04E-07	0.003782	6.317625
FOXS1	3.108929	7.947939	8.659109	6.45E-07	0.003782	6.259634
COL15A1	3.77972	6.222853	8.249504	1.13E-06	0.003782	5.757117
HS3ST1	2.703481	8.550209	8.22796	1.17E-06	0.003782	5.730045
KCNQ1	2.834809	8.175687	8.17903	1.25E-06	0.003782	5.668317
GLS2	-4.89212	8.213377	-8.09083	1.41E-06	0.003782	5.556202
PLGLB1	-4.36149	8.673836	-8.0758	1.44E-06	0.003782	5.536983
CDH11	3.121206	6.575013	8.006235	1.59E-06	0.003782	5.447624
GPR128	-3.08117	5.873277	-7.87956	1.91E-06	0.00402	5.283121
SLC44A2	2.90093	7.392111	7.773046	2.23E-06	0.00402	5.143004

Supplementary Table 6. Partial data used for the heat map and volcano map, which are shown in Figure 7.

miRNA	logFC	AveExpr	t	P.Value	adj.P.Val	B
hsa-miR-199b-5p	1.416778	5.601492	6.321877	5.33E-05	0.106904	1.576166
hsa-miR-1306-3p	-0.53955	4.498833	-5.45611	0.00019	0.190301	0.667024
hsa-miR-4711-3p	-0.19598	3.293092	-4.73714	0.00059	0.313941	-0.19265
hsa-miR-330-3p	0.323968	3.330821	4.700887	0.000626	0.313941	-0.23854
hsa-miR-4727-3p	-0.49023	3.542294	-4.4513	0.000945	0.315992	-0.56091
hsa-miR-4481	-0.86347	5.04559	-4.36131	0.001099	0.315992	-0.67986
hsa-miR-21-5p	1.940798	13.44862	4.359168	0.001103	0.315992	-0.68271
hsa-miR-23a-3p	1.32475	10.20463	4.276406	0.001268	0.317861	-0.79337
hsa-miR-99b-5p	1.457838	5.661595	4.107612	0.001689	0.320464	-1.02263
hsa-miR-383	-0.22044	3.274829	-4.02246	0.001955	0.320464	-1.14003

Supplementary Table 7. Partial data used for the heat map and volcano map, which are shown in Figure 8.

miRNA	logFC	AveExpr	t	P.Value	adj.P.Val	B
hsa-miR-182-5p	4.635451	-8.32478	4.205006	0.000964	0.073333	-0.60214
hsa-miR-141-5p	4.257346	-9.28875	4.087029	0.001207	0.08261	-0.81923
hsa-miR-96-5p	3.876296	-4.58163	3.554282	0.003372	0.144264	-1.81028
hsa-miR-135b-5p	3.774617	-3.34834	3.617931	0.00298	0.135977	-1.69131
hsa-miR-221-5p	3.51104	-8.96189	3.216891	0.00651	0.184768	-2.44049
hsa-miR-200c-3p	3.303703	-0.78762	5.609218	7.55E-05	0.015287	1.857204
hsa-miR-141-3p	3.096806	0.503038	5.589431	7.82E-05	0.015287	1.824462
hsa-miR-133a	2.916509	-9.99264	2.944093	0.011082	0.216474	-2.94601
hsa-miR-200a-3p	2.853824	0.024705	6.166488	2.96E-05	0.012558	2.754537
hsa-miR-429	2.846444	-2.40456	6.036198	3.67E-05	0.012558	2.549069

Supplementary Table 8. Partial data used for the heat map and volcano map, which are shown in Figure 9.

miRNA	logFC	AveExpr	t	P.Value	adj.P.Val	B
hsa-miR-451a	-2.79155	10.03294	-11.4952	4.89E-18	5.35E-15	30.65943
hsa-miR-187-5p	-1.24298	9.774973	-10.9533	4.63E-17	2.53E-14	28.45716
hsa-miR-195-5p	-2.30883	9.872653	-10.5473	2.54E-16	9.26E-14	26.7874
hsa-miR-1	-1.19837	9.010703	-10.2269	9.83E-16	2.69E-13	25.4586
hsa-miR-566	2.637499	12.442	10.00175	2.56E-15	5.60E-13	24.51965
hsa-miR-3120-3p	-1.01183	9.249268	-9.88415	4.22E-15	6.83E-13	24.02767
hsa-miR-130b-3p	-0.72228	9.605988	-9.84453	5.00E-15	6.83E-13	23.86169
hsa-miR-145-5p	-3.58445	11.05105	-9.79989	6.05E-15	6.83E-13	23.67456
hsa-miR-101-3p	-1.67609	9.324401	-9.79579	6.16E-15	6.83E-13	23.65738
hsa-miR-3197	2.196498	12.61633	9.77256	6.80E-15	6.83E-13	23.55993

Supplementary Table 9. Partial data used for the heat map and volcano map, which are shown in Figure 10.

lncRNA	logFC	AveExpr	t	P.Value	adj.P.Val
AC005550.3	1.6364344	-2.699809	3.425693415	0.00580161	0.400259503
AC016768.1	-0.65367	-3.37412	-2.22933654	0.04797486	0.438528511
AC068535.3	-1.651389	-3.844059	-2.44491353	0.03287971	0.426785395
AC092159.3	-0.73845	-0.991457	-2.87286841	0.01540249	0.407667987
AC099684.1	-0.634109	-3.771207	-2.75676339	0.0189292	0.407667987
AC104809.4	-0.954105	-5.04172	-2.68705323	0.02142236	0.407667987
AC132217.4	-1.118647	3.6700493	-2.71392257	0.02042491	0.407667987
ADORA2A-AS1	-1.763306	-2.986681	-2.35006429	0.03884782	0.429910777
AF131215.2	0.8215994	-5.341839	2.218198624	0.04891381	0.43948318
AF131215.3	0.8735371	-4.753748	2.743348025	0.01938546	0.407667987

Supplementary Table 10. Partial data used for the heat map and volcano map, which are shown in Figure 10.

miRNA	logFC	AveExpr	t	P.Value	adj.P.Val
hsa-miR-10b-5p	-0.956408693	9.936683356	-5.706773475	2.32E-07	2.57E-06
hsa-miR-125a-5p	-1.824751787	10.68652528	-8.212104985	5.66E-12	2.29E-10
hsa-miR-127-3p	-0.860799904	9.421909804	-5.892159791	1.09E-07	1.28E-06
hsa-miR-141-3p	-1.223498764	10.06599566	-4.347989522	4.38E-05	0.000277291
hsa-miR-143-5p	-0.82372149	9.554404984	-5.441789649	6.74E-07	6.58E-06
hsa-miR-144-3p	-0.9593441	9.17534841	-8.386951378	2.66E-12	1.12E-10
hsa-miR-145-5p	-3.584451532	11.05104708	-9.799887104	6.05E-15	6.83E-13
hsa-miR-200a-3p	-1.281155798	10.21173014	-4.33829334	4.54E-05	0.000283899
hsa-miR-200b-3p	-1.520142469	10.58453108	-3.634169022	0.00051608	0.002267434
hsa-miR-200c-3p	-1.523708226	10.62717915	-3.058778929	0.003107539	0.010333276

Supplementary Table 11. Partial data used for the heat map and volcano map, which are shown in Figure 10.

mRNA	logFC	AveExpr	t	P.Value	adj.P.Val
ABCA6	-3.390247733	9.903068297	-4.909588198	0.000246808	0.008869186
ABCA8	-2.169812358	5.973369157	-3.905808602	0.001652638	0.022740539
ACACB	-1.573924692	8.138055726	-4.867175919	0.000266838	0.009263157
ACADL	-3.375837068	7.172923236	-6.151065795	2.80E-05	0.005603656
ACOX2	-3.633032103	10.54599544	-5.563022309	7.64E-05	0.006555141
ACSL1	-2.259342462	11.0665055	-3.482538723	0.003781246	0.037582696
ACSS3	-3.028431074	8.207668847	-4.538725872	0.000491969	0.012075846
ADAMTS14	1.760100244	4.526309904	3.797717562	0.00203964	0.025938427
ADRA2B	-2.060357703	5.650165274	-4.277942057	0.000806697	0.015352334
AGTR1	-3.250938945	7.748404791	-4.845118311	0.000277915	0.009359392

Supplementary Table 12. Some of the results of the coexpression analysis and intersection of GSE61850 and GSE103909 datasets.

miRNA	lncRNA	Tot Score	Tot Energy	Max Score	Max Energy	mi_len	lnc_len
hsa-miR-10b-5p	PVT1	6805	-732.12	164	-24.21	23	306721
hsa-miR-10b-5p	RP11-434D9.1	4354	-490.26	163	-25.89	23	425861
hsa-miR-10b-5p	U91319.1	3936	-451.21	161	-23.47	23	316603
hsa-miR-10b-5p	THRB-AS1	2625	-282.95	159	-21.1	23	187625
hsa-miR-10b-5p	RP11-328K4.1	2352	-259.95	168	-23.15	23	197500
hsa-miR-10b-5p	AC016768.1	2189	-244.12	160	-23.9	23	180932
hsa-miR-10b-5p	RP11-767I20.1	1735	-208.32	154	-22.18	23	215789
hsa-miR-10b-5p	RP11-215P8.4	1717	-169.55	149	-19.81	23	31565
hsa-miR-10b-5p	LINC00937	1480	-169.41	157	-23.49	23	100818
hsa-miR-10b-5p	LINC01620	1584	-158.04	154	-22.6	23	29178

Supplementary Table 13. Some of results of the coexpression analysis and intersection of GSE57555 datasets.

miRNA	mRNA	r	p.value
hsa-miR-125a-5p	ABCA6	-0.53128	0.010947
hsa-miR-141-3p	ABCA6	-0.50615	0.016239
hsa-miR-143-5p	ABCA6	-0.54408	0.008855
hsa-miR-200a-3p	ABCA6	-0.61577	0.002282
hsa-miR-200b-3p	ABCA6	-0.65849	0.000862
hsa-miR-200c-3p	ABCA6	-0.53211	0.010801
hsa-miR-27a-3p	ABCA6	-0.49978	0.017865
hsa-miR-320b	ABCA6	-0.58921	0.003909
hsa-miR-338-3p	ABCA6	-0.43223	0.044541
hsa-miR-99b-5p	ABCA6	-0.59728	0.003335

Supplementary Table 14. Some of the results from a total of 29,426 miRNA-mRNA relationships predicted by the online tool mirwalk2.0.

miRNA	Gene
hsa-miR-10b-5p	GTF2H1
hsa-miR-10b-5p	PAFAH1B1
hsa-miR-10b-5p	TFAP2C
hsa-miR-10b-5p	FXR2
hsa-miR-10b-5p	ZMYND11
hsa-miR-10b-5p	SLC38A2
hsa-miR-10b-5p	ANK1
hsa-miR-10b-5p	COL4A4
hsa-miR-10b-5p	CREBL2
hsa-miR-10b-5p	ESRRG

Supplementary Table 15. Detailed results of 113 miRNA-mRNA total relationship pairs by intersecting with the miRNA-mRNA.

mi	mRNA	r	p.value
hsa-miR-125a-5p	ACACB	-0.42755	0.047167
hsa-miR-125a-5p	AR	-0.47674	0.024879
hsa-miR-125a-5p	PROS1	-0.50034	0.017716
hsa-miR-125a-5p	GFRA1	-0.56083	0.006622
hsa-miR-125a-5p	MOCS1	-0.48891	0.020942
hsa-miR-125a-5p	SERPINA5	-0.4716	0.02671
hsa-miR-125a-5p	C1RL	-0.54227	0.009128
hsa-miR-125a-5p	SFXN5	-0.5187	0.013384
hsa-miR-141-3p	MYRIP	-0.61345	0.002396
hsa-miR-141-3p	THRB	-0.49564	0.01899

Supplementary Table 16. Some of the results obtained for 362 miRNA-lncRNA relationship pairs that were predicted by using the local software Miranda (v3.3a).

miRNA	lncRNA	Tot Score	Tot Energy	Max Score	Max Energy
hsa-miR-10b-5p	PVT1	6805	-732.12	164	-24.21
hsa-miR-10b-5p	RP11-434D9.1	4354	-490.26	163	-25.89
hsa-miR-10b-5p	U91319.1	3936	-451.21	161	-23.47
hsa-miR-10b-5p	THRB-AS1	2625	-282.95	159	-21.1
hsa-miR-10b-5p	RP11-328K4.1	2352	-259.95	168	-23.15
hsa-miR-10b-5p	AC016768.1	2189	-244.12	160	-23.9
hsa-miR-10b-5p	RP11-767I20.1	1735	-208.32	154	-22.18
hsa-miR-10b-5p	RP11-215P8.4	1717	-169.55	149	-19.81
hsa-miR-10b-5p	LINC00937	1480	-169.41	157	-23.49
hsa-miR-10b-5p	LINC01620	1584	-158.04	154	-22.6

Supplementary Table 17. Some of the results from a total of 340 pairs of regulatory relationships that were used to construct the ICC-related ceRNETs.

Gene	mi	lncRNA
AOX1	hsa-miR-338-3p	AC104809.4
AOX1	hsa-miR-27a-3p	AC104809.4
AR	hsa-miR-125a-5p	AC104809.4
AR	hsa-miR-338-3p	AC104809.4
AR	hsa-miR-320b	AC104809.4
C1RL	hsa-miR-125a-5p	AC104809.4
C1RL	hsa-miR-320b	AC104809.4
C6	hsa-miR-320b	AC104809.4
CCBE1	hsa-miR-27a-3p	AC104809.4
CECR2	hsa-miR-27a-3p	AC104809.4

Supplementary Table 18. Some of the results of the upregulated and downregulated nodes in the constructed ICC-related ceRNETs.

lncRNA				
**lncRNA**	**GSE61850**	**GSE103909**	**up_down**	
RP11-328K4.1	-6.791	-2.115	down	
AC068535.3	-6.018	-1.651	down	
ADORA2A-AS1	-5.816	-1.763	down	
RP11-685F15.1	-4.589	-1.262	down	
FAM83A-AS1	-4.273	-0.999	down	
RP11-215P8.4	-3.985	-0.91	down	
LINC01485	-3.975	-2.214	down	
CTD-2527I21.4	-3.749	-1.173	down	
AC104809.4	-3.65	-0.954	down	
RP11-372H2.1	-3.57	-0.766	down	
**miRNA**				
**miRNA**	**GSE57555**	**GSE53992**	**GSE53870**	**up_down**
hsa-miR-10b-5p	1.3457	1.1616	-0.956	unsure
hsa-miR-125a-5p	1.1796	0.8644	-1.825	unsure
hsa-miR-127-3p	0.9055	0.4767	-0.861	unsure
hsa-miR-141-3p	2.4001	3.0968	-1.223	unsure
hsa-miR-143-5p	0.393	1.2037	-0.824	unsure
hsa-miR-144-3p	-2.3	-1.028	-0.959	down
hsa-miR-145-5p	0.8671	0.8803	-3.584	unsure
hsa-miR-200a-3p	1.4671	2.8538	-1.281	unsure
hsa-miR-200b-3p	1.8032	2.728	-1.52	unsure
hsa-miR-200c-3p	2.6416	3.3037	-1.524	unsure
**mRNA**				
**mRNA**	**GSE61850**	**GSE103909**	**GSE57555**	**up_down**
ABCA6	-4.474	-1.557	-3.39	down
ABCA8	-4.523	-1.243	-2.17	down
ACACB	-3.313	-0.917	-1.574	down
ACADL	-6.679	-2.234	-3.376	down
ACOX2	-5.668	-1.6	-3.633	down
ACSL1	-4.204	-1.478	-2.259	down
ACSS3	-4.242	-1.538	-3.028	down
ADRA2B	-3.571	-0.802	-2.06	down
AGTR1	-4.405	-1.669	-3.251	down
AKR1C4	-3.861	-2.596	-4.08	down

Supplementary Table 19. Partial results of key nodes in the constructed ICC-related ceRNETs.

node	degree	node_type		logFC	
ADORA2A-AS1	40	lnc_down	-5.816275703	-1.76330582	
RP11-328K4.1	40	lnc_down	-6.791185406	-2.11469899	
LINC01485	22	lnc_down	-3.975134266	-2.21436574	
RP4-639F20.1	21	lnc_down	-1.871698678	-0.70354943	
AC104809.4	18	lnc_down	-3.6500429	-0.95410501	
CTD-2527I21.4	18	lnc_down	-3.748693699	-1.17309379	
METTL7A	15	m_down	-3.526876688	-1.09831686	-2.76726
AR	13	m_down	-3.72196991	-1.2864071	-3.22778
CECR2	13	m_down	-3.906689217	-1.2685956	-1.2702
PROS1	12	m_down	-3.407107204	-1.22277576	-2.534
C6	11	m_down	-6.206844508	-2.80975279	-4.82322
IVD	11	m_down	-1.565671255	-0.89605639	-1.74028
hsa-miR-200a-3p	26	mi_unsure	1.467072735	2.853823558	-1.28116
hsa-miR-27a-3p	25	mi_unsure	1.178734196	1.201162186	-2.96797
hsa-miR-200c-3p	23	mi_unsure	2.641633829	3.303703476	-1.52371
hsa-miR-141-3p	22	mi_unsure	2.40005083	3.09680579	-1.2235
hsa-miR-200b-3p	20	mi_unsure	1.803157787	2.727988339	-1.52014
hsa-miR-125a-5p	19	mi_unsure	1.179603522	0.864363512	-1.82475
